# Broad-Spectrum Protective Effects of Lyophilized FE002-Lu Lung Fibroblast Conditioned Medium Against Acute and Chronic Pulmonary Injury in Wistar Rats

**DOI:** 10.3390/biomedicines14091888

**Published:** 2026-08-24

**Authors:** Lee Ann Applegate, Alexandre Porcello, Alexis E. Laurent

**Affiliations:** 1 Manufacturing Department, ELANIX Sàrl, CH-1038 Bercher, Switzerland; 2 Center for Applied Biotechnology and Molecular Medicine, University of Zurich, CH-8057 Zurich, Switzerland; 3 Oxford OSCAR Suzhou Center, Oxford University, Suzhou 215123, China; 4 Development Department, Abcello Sàrl, CH-1266 Duillier, Switzerland; alexandre.porcello@abcello.com; 5 Manufacturing Department, TEC-PHARMA SA, CH-1038 Bercher, Switzerland; 6 Manufacturing Department, LAM Biotechnologies SA, CH-1066 Epalinges, Switzerland

**Keywords:** anti-fibrotic, cytokine storm, extracellular matrix, induced lung injury, intratracheal administration, myofibroblast activation, pulmonary fibrosis, pulmonary inflammation

## Abstract

**Background**: While live-cell therapies face significant translational hurdles, cell-free secretomes derived from fetal progenitor cells offer a unique, scalable approach natively programmed for scarless tissue repair. **Methods**: This GLP-compliant study evaluated the therapeutic efficacy spectrum of an off-the-shelf, clinical-grade, intratracheal lyophilized FE002-Lu lung fibroblast conditioned medium (LFCM) across five controlled Wistar rat models of induced lung injury: bleomycin (5 mg/kg), asbestos (100 µg/rat), hyperoxia (100% oxygen exposure), silica (30 mg/rat), and lipopolysaccharide (LPS, 4 mg/kg). Following lung injury induction and symptom onset, symptomatic rats were randomized to receive intratracheal LFCM (low, mid, or high dose) or a vehicle control every 4 days for 28 days. **Results**: The intervention demonstrated an exceptional safety profile, maintaining 100% survival with no severe procedural toxicity across all cohorts. Across all study arms, LFCM effectively attenuated pulmonary inflammation, reducing pro-inflammatory markers (IL-1β, IL-6, TNF-α, and CINC-1) in bronchoalveolar lavage fluid. Concurrently, high LFCM doses consistently elevated the anti-inflammatory cytokine IL-10. Within the lung tissue, mid- and high doses significantly reduced key pro-fibrotic drivers, including TGF-β1, TIMP-1, WISP-1, and hydroxyproline. Treatments consistently decreased α-SMA expression and pro-fibrotic gene mRNA levels, mitigating pulmonary myofibroblast activation. This correlated with reduced Ashcroft scores and collagen deposition, thereby preserving lung architecture. Notably, the FE002-Lu LFCM treatment exerted a biphasic regulation of extracellular matrix turnover: it elevated Cathepsin-D and MMP-12 in the bleomycin arm to actively clear newly deposited fibrotic debris, while reducing these markers in the hyperoxia and LPS arms to prevent acute collateral degradation of the native lung matrix. In both particulate models (asbestos and silica), the LFCM mid-dose established an optimal therapeutic threshold, avoiding the localized secretome saturation and pro-fibrotic exacerbation occasionally observed at higher doses. **Conclusions**: Lyophilized FE002-Lu LFCM acts as a potent, pleiotropic biologic that effectively resolves acute pulmonary inflammation and arrests progressive fibrotic remodeling across multiple distinct in vivo models. By overcoming the cold-chain and delivery limitations inherent to pulmonary live-cell therapies, this stable, cell-free secretome represents a highly scalable, “off-the-shelf” candidate poised for non-invasive, aerosolized clinical translation.

## 1. Introduction

Acute and chronic lung injuries (LIs), encompassing a heterogeneous clinical spectrum ranging from acute respiratory distress syndrome (ARDS) to progressive interstitial lung diseases (ILDs) such as idiopathic pulmonary fibrosis (IPF), represent a massive and escalating global health burden [1,2]. The fundamental pathophysiology underlying these conditions is the severe and persistent disruption of the delicate pulmonary epithelial and endothelial barrier architecture [3,4]. This structural collapse precipitates a rapid cascade of deleterious molecular events: initial alveolar flooding with protein-rich exudate and significant extravasation of polymorphonuclear neutrophils, followed by an unchecked, self-amplifying local immune response often characterized as a cytokine storm [2,3,4,5].

When homeostatic resolution mechanisms fail, the alveolar microenvironment shifts rapidly toward a pro-fibrotic state. This aberrant wound healing is orchestrated by key fibrogenic drivers, notably transforming growth factor-beta 1 (TGF-β1), which promotes the transdifferentiation of resident fibroblasts into α-smooth muscle actin (α-SMA)-expressing myofibroblasts [6]. The resulting pathological accumulation of extracellular matrix (ECM) proteins, such as collagen and hydroxyproline, leads to irreversible structural remodeling and catastrophic loss of gas exchange capacity [2,4,6,7]. Despite extensive advances in our understanding of pulmonary cellular crosstalk, the available clinical armamentarium remains highly limited. Current single-target pharmacotherapies primarily offer supportive care or merely decelerate fibrotic progression, failing to halt the underlying structural degradation or significantly reduce mortality rates [6,8]. Consequently, identifying highly scalable, pleiotropic therapeutic strategies capable of simultaneously mitigating acute inflammatory cascades and suppressing chronic fibrotic pathways across diverse etiologies remains a critical, unmet imperative in respiratory medicine.

In recent years, the paradigm of regenerative medicine has fundamentally shifted away from live-cell engraftment toward the use of cell-free therapeutics [9,10]. It is now widely recognized that the significant regenerative, immunomodulatory, and anti-fibrotic benefits of stem and progenitor cells are predominantly mediated via their paracrine factors rather than direct cellular replacement [9,10]. Conditioned medium captures the complex, bioactive cell secretome, a highly concentrated milieu of soluble cytokines, growth factors, and target-modulating extracellular vesicles (EVs) [10]. Utilizing the secretome as a drug substance circumvents the inherent limitations of live-cell therapies, notably mitigating the risks of post-implantation cellular senescence, pulmonary entrapment, alloimmunization, and tumorigenicity [9,11,12]. Furthermore, beyond its superior biological safety profile, the cell-free nature of this isolated secretome allows for advanced pharmaceutical stabilization. In the present study, the investigated FE002-Lu progenitor lung fibroblast conditioned medium (LFCM) is formulated as a lyophilized powder within a sterile 2R vial (i.e., “off-the-shelf” biologic) [13]. Importantly, integrating the lyophilization process circumvents the reliance on stringent cryopreservation and complex cold-chain logistics, which are notorious bottlenecks that historically restrict the clinical translation, patient accessibility, and commercial viability of advanced regenerative therapies [11,13,14].

Biologically, the current investigation leverages the FE002 cell source, which provides an exceptionally robust, clinical-grade platform for generating cell-based and cell-derived therapeutic materials. Established from a single human organ donation in Switzerland (2009) under strict regulatory and ethical frameworks, each of the tissue-specific variants of the FE002 fetal progenitor cell source bypasses the significant batch-to-batch variability, epigenetic drift, and donor-dependent inconsistencies that plague adult mesenchymal stem cell (MSC) therapies [15]. Primary fetal progenitor cells (FPCs) occupy a highly advantageous ontogenetic niche: they possess inherent biological superiority, including robust telomerase activity, superior genomic stability, high biosafety profiles, and a developmental priming for rapid organogenesis and scarless tissue repair [15]. The specific diploid cell type utilized in this GLP-compliant study, FE002-Lu, consists of cultured primary fetal lung fibroblasts natively programmed to orchestrate pulmonary microenvironment homeostasis, notably via matrix metalloproteinases (MMPs) and tissue inhibitors of metalloproteinases (TIMPs) [15,16]. In detail, based on our aggregated prior preclinical and clinical evidence around dermal progenitor cells (FE002-SK2 cells) from the same biological source, the FE002-Lu LFCM secretome appeared as a prime candidate for local, tissue-specific preventive or therapeutic applications in the lung [17,18,19].

To comprehensively evaluate the therapeutic spectrum of such a pleiotropic intervention, diverse and parallel experimental in vivo models are necessary to accurately simulate the multifaceted pathophysiological triggers of clinical LI [3,20,21,22,23,24]. Indeed, the drivers of pulmonary failure are vast, encompassing oxidative, infectious, occupational, and pharmacological hazards [1,24,25]. Hyperoxia, while a critical and ubiquitous life-saving intervention for acute hypoxemia, rapidly induces direct endothelial toxicity and early oxidative damage to lung tissues [2,26,27,28]. The massive generation of reactive oxygen species (ROS) overwhelms endogenous antioxidant defenses, leading to lipid peroxidation, mitochondrial dysfunction, and early alveolar type II cell apoptosis, often significantly preceding overt functional decline [26,27,28,29]. Conversely, infectious triggers, such as lipopolysaccharides (LPSs), simulate severe Gram-negative bacterial pneumonia. Therein, LPS acts as a potent endotoxin, binding to Toll-like receptor 4 (TLR4) to initiate a robust, multi-stage acute phase response via nuclear factor-κB (NF-κB) translocation, culminating in massive alveolar permeability and the pathological elevation of pro-inflammatory cytokines such as IL-1β, IL-6, and TNF-α [5,30,31,32,33,34].

Furthermore, occupational exposures to airborne pathogenic particulates remain a sentinel cause of irreversible respiratory disease globally. Specifically, asbestos fibers and silica crystals present unique aerodynamic profiles that allow deep alveolar deposition [35]. Once deposited, resident alveolar macrophages attempt clearance, leading to frustrated phagocytosis, lysosomal membrane permeabilization, and subsequent chronic activation of the NLRP3 inflammasome. This persistent localized oxidative stress drives continuous macrophage activation, compensatory epithelial hyperplasia, and a chronic inflammatory state that frequently evolves into fatal fibroproliferative diseases such as asbestosis and silicosis [35,36,37,38].

Finally, controlled pharmacological models are widely used for understanding the terminal stages of fibrotic progression. Bleomycin, a chemotherapeutic agent known to induce single- and double-strand DNA breaks, reliably triggers alveolar epithelial cell necrosis and senescence [39,40,41,42]. The resulting damage heavily upregulates TGF-β1 and WNT signaling pathways (e.g., WISP-1), driving progressive, chronic pulmonary fibrosis characterized by excessive collagen deposition and contractile myofibroblast hyperproliferation, making this model the gold standard for studying IPF [6,42,43].

Importantly, while individual in vivo lung injury models have been extensively characterized, evaluating a single standardized therapeutic approach across this entire mechanistic spectrum simultaneously is uncommon [24,44,45]. Thus, the primary novelty and scientific significance of this preclinical study lie in its comprehensive, parallel translational investigation of a highly standardized, off-the-shelf biological complex across five fundamentally distinct mechanisms of in vivo lung damage (hyperoxia, LPS, asbestos, silica, and bleomycin toxicants). By eliminating the inter-study variability that typically confounds the evaluation of novel biological therapeutics, this robust GLP-compliant, multi-arm design provides a broad and highly controlled assessment of FE002-Lu LFCM’s widespread applicability, optimal dosing regimens, and molecular mechanisms of action. Overall, the primary objective of this study was to establish the broad-spectrum in vivo functional proof-of-concept (rigorous multi-etiology biological baseline) and the safety profile of FE002-Lu LFCM across diverse etiologies of LI in Wistar rats, before moving to further complex secretome characterization and subsequent preclinical development.

Therefore, we hypothesize that the repeated intratracheal administration of lyophilized, tissue-specific FE002-Lu LFCM will locally exert potent, dose-dependent, and broad-spectrum beneficial effects regardless of the initial LI etiology. We further postulate that this highly scalable, cell-free biological therapy prototype will actively suppress acute pro-inflammatory cascades, stabilize lysosomal and ECM dynamics, and effectively mitigate myofibroblast activation. Through these multifaceted mechanisms, we anticipate that FE002-Lu LFCM will work toward halting fibrotic progression, preserve lung architecture, and transition the pulmonary microenvironment from a state of progressive injury toward sustained structural and functional resolution.

## 2. Materials and Methods

### 2.1. Lyophilized LFCM Preparation and Control

#### 2.1.1. Cell Source and In Vitro Expansion

Primary fetal lung progenitor fibroblasts (FE002-Lu cell type) were originally procured and cultured under the strict regulatory framework of the Swiss progenitor cell transplantation program [15]. Starting cells for this study were supplied as a fully characterized Working Cell Bank (WCB) from an internal international mirror biorepository (Shandong Province, China). All clinical-grade parental cell stocks were originally derived from a thoroughly documented single-donor source, analytically and medically confirmed free of intrinsic and adventitious contaminants, and maintained in liquid nitrogen vapor phase in Good Manufacturing Practice (GMP)-compliant biorepositories. For FE002-Lu cellular expansion, the contents of the WCB cryovials were thawed and seeded in high-glucose Dulbecco’s Modified Eagle Medium (DMEM, BioEngine, Shanghai, China) supplemented with GMP-grade fetal bovine serum (FBS, JYK, Hohhot, China) and L-glutamine (Shanghai Kyowa, Shanghai, China). The cultures were maintained in a humidified incubator system at 37 °C under a normoxic, 5% CO_2_ atmosphere.

#### 2.1.2. Secretome Harvest and Processing

Cells were serially expanded in vitro to yield confluent monolayers at passage level 8. To initiate secretome collection, the FE002-Lu monolayers were washed three times with sterile Dulbecco’s Phosphate-Buffered Saline (DPBS, BioEngine, Shanghai, China) to thoroughly eliminate residual FBS and culture medium components. The culture vessels were then replenished with basal DPBS and incubated overnight. The resulting FE002-Lu secretome-enriched DPBS (LFCM) was harvested, pooled, and centrifuged at 2900× *g* for 20 min to pellet cellular debris and apoptotic bodies. The resulting solution was normalized by dilution/concentration to reach 50 ± 5 µg/mL total protein, as determined by a BCA assay. To ensure strict sterility, the clarified supernatant was sequentially filtered through polyethersulfone (PES) membranes of 0.45 µm and 0.22 µm porosity.

#### 2.1.3. Secretome Formulation and Lyophilization

To formulate the final bulk drug substance, the sterile filtrate was supplemented with a stabilizing cryo- and lyoprotective excipient blend consisting of equimolar concentrations of parenteral-grade sucrose (AVT, Shanghai, China) and trehalose (AVT, Shanghai, China). Under Grade A laminar airflow cleanroom conditions, aliquots of the formulated bulk were dispensed into sterile 2R Type I borosilicate glass lyophilization vials (Taian Youlyy, Taian, China). The vials were subjected to a controlled-rate freezing profile, cooling at −1.5 °C/min from ambient temperature to a final setpoint of −55 °C with annealing. The lyophilization cycle was conducted overnight under a vacuum of 190 mTorr. Following drying, the vials were stoppered in situ under a chemically inert, anhydrous atmosphere (medical-grade argon, Air Liquide China, Shanghai, China) and hermetically sealed. Finished LFCM batches were labeled, boxed, and securely stored at 4 °C or shipped at ambient temperature for further use.

#### 2.1.4. Quality Control and Batch Release

To ensure pharmaceutical-grade batch consistency and safety, the manufactured lyophilized LFCM underwent rigorous post-production quality control protocols. These evaluations included:

- Descriptive Controls: Standardized operator grading of the lyophilized cake architecture and attributes.
- Quality Analytics: Uniformity of mass, Karl Fischer titration for residual moisture, and BCA assays for total protein quantification.
- Safety Testing: Pharmacopeial sterility testing, visible and sub-visible particulate counts, and Limulus amebocyte lysate (LAL) endotoxin testing.
- Functional Assays: Validated Trolox Equivalent Antioxidant Capacity (TEAC) assays and reporter cell line assays to confirm retained activity.

### 2.2. Animal Husbandry and Experimental Design

#### 2.2.1. Animals and Housing Conditions

All animal experiments were conducted at Trans-Genica Services, Maharashtra, India, under protocol N°TRS/PT/025/065, approved on 4 April 2025 by the Institutional Animal Ethics Committee, pursuant to registration by the Committee for Control and Supervision of Experiments on Animals. The animal-related experiments of the study were all GLP-compliant. Healthy, young adult male Wistar rats (i.e., *n* = 40 per study arm; aged 9–11 weeks; approximate body weight of 150 g at receipt) were procured for the development of the five distinct lung injury models (five study arms). The specific scientific rationale and study limitations regarding the exclusive use of male animal subjects to establish a highly susceptible, non-estrogen-protected fibrotic baseline are detailed in Section 4.7. Animals were housed under standardized, environmentally controlled conditions (temperature: 22 ± 3 °C; relative humidity: 50 ± 20%; 12 h light/dark cycle). The rats were group-housed (three animals per cage) in sterile polypropylene cages (427 × 287 × 198 mm) lined with dust-free, pure corncob bedding to maintain optimal hygiene. Standard laboratory pelleted rodent diet and purified, filtered drinking water were provided ad libitum. Cages, bedding, and water bottles were replaced according to standard institutional husbandry protocols.

#### 2.2.2. Acclimatization and Baseline Health Assessment

Upon arrival, all animals underwent a mandatory five-day acclimatization period prior to toxicant exposure. During this phase, animals were monitored once daily for general clinical signs, morbidity, and mortality. A comprehensive veterinary examination was conducted on each individual animal prior to study inclusion to ensure baseline health and suitability for experimental procedures.

#### 2.2.3. Lung Injury Induction Screening and Randomization

Following toxicant exposure/administration, all animals were prospectively screened to confirm the successful induction of LI prior to therapeutic intervention. Successful induction was validated by the onset of established clinical signs of respiratory distress and systemic inflammation. These criteria included pronounced abdominal breathing, chromodacryorrhea (porphyrin secretion/red tears), nasal discharge, epistaxis, peripheral erythema, and audible respiratory stridor or stertor indicative of mucosal edema and exudative accumulation.

#### 2.2.4. Group Allocation and Dosing Regimen

Animals demonstrating successful disease induction were randomized into five experimental groups (*n* = 8 per group) for each distinct study arm. Appropriate group size was prospectively determined via an a priori power analysis. Based on the expected variance in our primary structural endpoint (Ashcroft fibrosis scores), we estimated a conservative standard deviation of approximately 1.0 unit. To reliably detect a minimal biologically significant mean difference of 1.5 units between the vehicle-treated disease control and the therapeutic cohorts, with a predefined Type I error rate (α) of 0.05 and a desired statistical power (1–β) of 80%, a minimum of 7 animals per group was required. The final sample size was established at *n* = 8 rats per group to account for potential baseline disease- or procedure-related attrition, ensuring robust statistical powering at the study’s conclusion.

The experimental cohorts were defined as follows:

- Group 1 (G1; Sham Control): Received no toxicant and was administered a sterile saline vehicle.
- Group 2 (G2; Positive/Disease Control): Received the toxicant followed by the administration of a vehicle control (no therapeutic treatment).
- Groups 3, 4, and 5 (G3–G5; Treatment Groups): Received the toxicant followed by the administration of FE002-Lu LFCM at low, mid, and high doses, respectively.

For the therapeutic interventions, the high-dose treatment (G5) consisted of the undiluted, reconstituted LFCM, representing the maximal soluble concentration of the FE002-Lu secretome (normalized to 50 ± 5 µg/mL total protein). Because this study was designed as the foundational in vivo dose-finding investigation for this biologic, prior in vivo optimization studies were not conducted. Instead, to accurately map a broad pharmacological dose–response curve, the mid-dose (G4) and low-dose (G3) treatments were systematically prepared as twofold (1:2) and fourfold (1:4) serial dilutions of the maximal reconstituted LFCM, respectively. All treatments and vehicle controls were administered via the intratracheal (i.t.) route at a standardized volume of 50 µL per defined timepoint for each animal.

### 2.3. Lung Injury Development

#### 2.3.1. Hyperoxia-Induced Lung Injury Model

Controlled hyperoxic exposures were conducted utilizing a custom-fabricated acrylic inhalation chamber (30 × 20 × 15 cm). The chamber was continuously infused with 100% medical-grade oxygen (i.e., fraction of inspired oxygen, FiO_2_ = 1.0) delivered via a reinforced silicone-conduit tubing system connected to a certified compressed oxygen cylinder (Harshita Gases, Jalgaon, India). To serve as a baseline, sham control animals were housed in identical chambers but maintained under standard normoxic ambient air conditions (FiO_2_ = 0.21).

The hyperoxia-induced LI was established using a biphasic exposure regimen. Initially, animals were subjected to continuous 100% oxygen exposure within the closed chamber for five consecutive days. To further exacerbate and consolidate the LI, this initial continuous phase was followed by a repetitive exposure protocol. During this secondary phase, animals were subjected to 100% oxygen for 120 min daily over 10 consecutive days. Following each intermittent exposure session, the rats were immediately returned to their standard home cages under normoxic conditions with ad libitum access to food and water.

Successful induction of the hyperoxic LI typically manifested within 8 to 10 days from the onset of the exposure period. Throughout the protocol, animals were comprehensively evaluated twice daily for clinical manifestations of pulmonary toxicity and systemic distress. The monitored parameters included pronounced abdominal breathing, chromodacryorrhea (porphyrin secretion), mucoid nasal discharge, epistaxis, piloerection, peripheral erythema (indicative of systemic inflammation), and audible respiratory stertor or stridor secondary to mucosal edema and exudate accumulation. These clinical signs were systematically graded on a standardized severity scale (mild, moderate, or severe). Following the completion of the exposure protocol, symptomatic animals were stratified based on their cumulative clinical severity scores and randomized into their respective experimental cohorts, ensuring a uniform baseline disease burden across all treatment groups prior to therapeutic intervention.

#### 2.3.2. Lipopolysaccharide-Induced Lung Injury Model

Lipopolysaccharide (LPS) derived from *Escherichia coli* (*E. coli*, serotype O55:B5, Sigma-Aldrich, St. Louis, MO, USA) was reconstituted in sterile physiological saline (0.9% NaCl) to yield a clear, fully solubilized working solution. The prepared formulation was protected from light and stored at 2–8 °C. Immediately prior to i.t. administration, the required aliquots were allowed to equilibrate to ambient temperature for 2 to 3 min.

To induce acute pulmonary injury, animals were anesthetized. Following the confirmation of a deep surgical plane of anesthesia, a single 4 mg/kg dose of the LPS formulation was administered via the i.t. route. Instillation was carefully performed through the glottal opening utilizing a 14G or 16G endotracheal tube coupled to a 1 mL tuberculin syringe (Terumo, Tokyo, Japan). Sham control animals underwent an identical procedural protocol but received an equivalent volume (0.1 mL) of sterile saline vehicle. To facilitate homogeneous bilateral pulmonary distribution of the toxicant and maintain airway patency, animals were positioned at a 45° upright incline during and immediately following the dosing procedure.

Following instillation, animals were placed on a thermostatically controlled heating pad to maintain normothermia until full recovery from the anesthesia. They were subsequently returned to their standard home cages with ad libitum access to food and water. The acute LPS-induced LI phenotype successfully manifested within 7 to 9 days post-instillation. During this developmental window, animals were rigorously monitored twice daily for clinical indicators of acute respiratory distress and systemic endotoxemia. Assessed parameters included pronounced abdominal breathing, chromodacryorrhea (porphyrin secretion), mucoid nasal discharge, epistaxis, piloerection, gasping (open-mouth agonal respiration), peripheral erythema (indicative of systemic inflammation), and audible respiratory stertor or stridor secondary to mucosal edema and exudative pooling.

Clinical manifestations were systematically documented and graded on a standardized severity scale (mild, moderate, or severe). Following the completion of the 7- to 9-day induction phase, symptomatic animals were stratified based on their cumulative clinical severity scores and randomized into their respective experimental cohorts, ensuring a uniform baseline disease burden across all groups prior to therapeutic intervention.

#### 2.3.3. Asbestos-Induced Lung Injury Model

Chrysotile asbestos fibers (approximate particle size: 5 μm, Pearl Industries, Delhi, India) were homogeneously suspended in sterile physiological saline (0.9% NaCl) to yield a turbid particulate suspension. The formulation was maintained at ambient temperature and thoroughly agitated immediately prior to administration to prevent particulate sedimentation and ensure uniform dosing.

To induce fibro-inflammatory pulmonary injury, animals were anesthetized. Following the confirmation of a deep surgical plane of anesthesia, a single 100 μg dose of the chrysotile asbestos suspension was administered via the i.t. route. Instillation was performed using the same precautions and protocol as for the LPS arm.

Following toxicant instillation, animals were placed on a thermostatically controlled heating pad to maintain normothermia until full recovery from the anesthesia. They were subsequently returned to their standard home cages with ad libitum access to food and water. The asbestos-induced LI phenotype successfully manifested within 10 to 12 days post-instillation. During this developmental window, animals were rigorously monitored twice daily for clinical indicators of acute respiratory distress and early fibroproliferative inflammation. Assessed parameters were the same as in the LPS arm.

Clinical manifestations were systematically documented and graded on a standardized severity scale (mild, moderate, or severe). Following the completion of the 10- to 12-day induction phase, symptomatic animals were stratified based on their cumulative clinical severity scores and randomized into their respective experimental cohorts, ensuring a uniform baseline disease burden across all groups prior to therapeutic intervention.

#### 2.3.4. Silica-Induced Lung Injury Model

Crystalline silica (silicon dioxide, SiO_2_; approximate particle size: 1–5 μm, Research-Lab Fine Chem Industries, Mumbai, India) was homogeneously suspended in sterile physiological saline (0.9% NaCl). Because silica particulates rapidly sediment in solution, the formulation was maintained at ambient temperature and vigorously dispersed using a vortex mixer immediately prior to each i.t. administration to ensure highly uniform dosing.

To induce fibrotic pulmonary injury, animals were anesthetized. Following the confirmation of a deep surgical plane of anesthesia, a single 30 mg dose of the silica suspension was administered via the i.t. route on day 0. Instillation was performed using the same precautions and protocol as for the LPS arm.

Following toxicant instillation, animals were placed on a thermostatically controlled heating pad to maintain normothermia until full recovery from the anesthesia. They were subsequently returned to their standard home cages with ad libitum access to food and water. The silica-induced LI phenotype (silicosis model) successfully manifested within 10 to 12 days post-instillation. During this developmental window, animals were rigorously monitored twice daily for clinical indicators of acute respiratory distress and early fibroproliferative inflammation. Assessed parameters were the same as in the LPS arm.

Clinical manifestations were systematically documented and graded on a standardized severity scale (mild, moderate, or severe). Following the completion of the 10- to 12-day induction phase, symptomatic animals were stratified based on their cumulative clinical severity scores and randomized into their respective experimental cohorts, ensuring a uniform baseline disease burden across all groups prior to therapeutic intervention.

#### 2.3.5. Bleomycin-Induced Lung Injury Model

Bleomycin sulfate (Tokyo Chemical Industry, Adambakkam, India), a well-characterized chemotherapeutic and pro-fibrotic agent, was fully solubilized in sterile physiological saline (0.9% NaCl). The prepared formulation was protected from light, stored at 2–8 °C to preserve its stability, and allowed to equilibrate to ambient temperature for 2 to 3 min immediately prior to i.t. administration.

To induce fibrotic pulmonary injury, animals were anesthetized. Following the confirmation of a deep surgical plane of anesthesia, a single 5 mg/kg dose of the bleomycin sulfate solution was administered via the i.t. route. Instillation was performed using the same precautions and protocol as for the LPS arm.

Following toxicant instillation, animals were placed on a thermostatically controlled heating pad to maintain normothermia until full recovery from the anesthesia. They were subsequently returned to their standard home cages with ad libitum access to food and water. The acute early-phase bleomycin-induced LI phenotype successfully manifested within 5 to 7 days post-instillation. During this developmental window, animals were rigorously monitored twice daily for clinical indicators of acute respiratory distress and early fibroproliferative inflammation. Assessed parameters were the same as in the LPS arm.

Clinical manifestations were systematically documented and graded on a standardized severity scale (mild, moderate, or severe). Following the completion of the 5- to 7-day induction phase, symptomatic animals were stratified based on their cumulative clinical severity scores and randomized into their respective experimental cohorts, ensuring a uniform baseline disease burden across all groups prior to therapeutic intervention.

### 2.4. Intratracheal Administration Procedure and LFCM Dosing Regimen

#### 2.4.1. Anesthesia Procedure and Animal Positioning

To ensure procedural safety and minimize animal distress, rats were anesthetized via intraperitoneal (i.p.) injection of a standardized cocktail containing ketamine hydrochloride (60 mg/kg, Troikaa Pharmaceuticals, Ahmedabad, India) and xylazine hydrochloride (10 mg/kg, Biograde Organics, Punjab, India). Once a deep surgical plane of anesthesia was confirmed (i.e., verified by the loss of the pedal withdrawal reflex), each animal was securely restrained in a supine position on a custom intubation board. The board was inclined at a 45° angle with the cranial aspect elevated to optimize orotracheal alignment and facilitate the gravitational delivery of the administered fluid to the lower airways.

#### 2.4.2. Intratracheal Instillation Technique

Intratracheal intubation was meticulously performed in aseptic conditions under direct transoral visualization. The tongue was gently retracted laterally to expose the oropharynx, and a veterinary otoscope equipped with a standard ear speculum was introduced to elevate the epiglottis and clearly visualize the glottic opening. A semi-rigid guiding stylet was then carefully advanced through the vocal cords into the proximal trachea. A modified 14G or 16G blunt-ended dosing cannula (approximately 5 cm in length, Terumo, Tokyo, Japan), attached to a 1 mL tuberculin syringe, was advanced over the stylet into the tracheal lumen. Following the withdrawal of the stylet, the target solution (toxicant, LFCM, or sterile vehicle) was slowly instilled. To ensure homogeneous bilateral pulmonary liquid distribution, ease animal breathing, and prevent reflex aspiration or instillate reflux, animals were maintained in the 45° elevated position (with the head facing upward) during and immediately following the procedure. After instillation, the cannula was gently removed, and animals were monitored until full recovery from anesthesia.

#### 2.4.3. Therapeutic Dosing Regimen

Following the successful induction of LI and subsequent group randomization (designated as day 0 of the treatment phase), the chronic therapeutic intervention protocol was initiated. Animals in the specific treatment cohorts received a standardized fixed volume of 50 µL of LFCM per administration. To comprehensively evaluate therapeutic efficacy and sustained tissue remodeling, the LFCM treatment protocol spanned a 28-day duration. Each animal received a total of eight successive LFCM i.t. administrations. These doses were systematically administered every four days (maintaining a strict 3-day recovery interval between successive doses to minimize the cumulative impact of anesthesia) on days 0, 4, 8, 12, 16, 20, 24, and 28 of the treatment phase. Negative control groups underwent an identical procedural regimen but received a sterile saline vehicle (Religrace Parenteral, Dewas, India).

Of note, while the lyophilized FE002-Lu LFCM was formulated with an equimolar lyo/cryoprotective blend of sucrose and trehalose, sterile physiological saline was selected as the vehicle control for the sham and disease cohorts. This specific design choice was implemented to establish an unadulterated, universally recognized physiological baseline for acute pulmonary injury and fibrotic progression. Both sucrose and trehalose are excipients that are standard components in approved inhalation therapies. At the trace concentrations present in the 50 µL reconstituted LFCM dose, these disaccharides function strictly to ensure biological component structural stabilization during the lyophilization cycle. While they do not possess independent, disease-modifying pharmacological activity, establishing the injury baseline with physiological saline ensures that the structural and molecular shifts observed in the treatment cohorts can be confidently attributed to the complex bioactive secretome rather than baseline excipient interactions.

### 2.5. Clinical Observations and Terminal Sacrifice

#### 2.5.1. Clinical Monitoring and Body Weight Assessment

Throughout the entire experimental duration, all animals were rigorously monitored twice daily for morbidity, mortality, and the onset of any adverse clinical manifestations. Comprehensive physical examinations were conducted to assess parameters indicative of respiratory distress and systemic toxicity. These evaluated parameters included pronounced abdominal breathing, chromodacryorrhea (porphyrin secretion), mucoid nasal discharge, epistaxis, piloerection, gasping (agonal respiration), peripheral erythema, and audible indicators of mucosal exudate accumulation. All observed clinical signs were systematically documented and graded according to a standardized severity scale. To continuously monitor the gross physiological health and nutritional status of the cohorts, individual animal body weights were recorded immediately prior to each i.t. administration on days 0, 4, 8, 12, 16, 20, 24, and 28 of the treatment phase, with a final body weight recorded on day 29.

#### 2.5.2. Euthanasia Protocol

Upon the successful completion of the 28-day therapeutic intervention period, all surviving animals in the study cohorts were humanely euthanized on day 29. Euthanasia was achieved via an i.p. administration of a lethal overdose of sodium thiopental (125 mg/kg, Neon Laboratories, Mumbai, India). This procedure was conducted in strict adherence to approved institutional animal care guidelines to ensure a rapid and painless death prior to the scheduled downstream tissue harvest and biological sampling. Blood samples were collected in EDTA tubes for total cell counts.

### 2.6. Bronchoalveolar Lavage Fluid Assessment

#### 2.6.1. BALF Collection and Processing

Immediately following euthanasia, the trachea was surgically exposed and cannulated in situ using a sterile airway catheter. Bronchoalveolar lavage was performed by gently instilling a standardized 5 mL volume of sterile physiological saline into the pulmonary tree. The fluid was then carefully aspirated to maximize recovery while minimizing shear stress and alveolar capillary rupture. The total recovered bronchoalveolar lavage fluid (BALF) volume was precisely recorded for each animal.

#### 2.6.2. Cellular Profiling

To assess acute inflammatory cell infiltration into the alveolar space, a 0.5 mL aliquot of the raw, uncentrifuged BALF was immediately diverted for cellular analysis. This aliquot was processed using an automated veterinary hematology analyzer (Mindray Vet-BC4000, Mindray, Shenzhen, China) to determine the total white blood cell (WBC) count alongside a comprehensive differential leukocyte profile, specifically quantifying lymphocytes, neutrophils, and eosinophils, as well as circulating platelets. Blood cellular profiles were determined similarly.

#### 2.6.3. Biomarker and Cytokine Quantification

The remaining BALF volume was subjected to centrifugation at 500× *g* for 7 min at 4 °C to cleanly pellet the cellular fraction and debris. The resulting cell-free supernatant was carefully collected, aliquoted to avoid repeated freeze–thaw cycles, and frozen at −20 °C until downstream biochemical evaluation. The concentrations of critical pro- and anti-inflammatory mediators (i.e., IL-1β [Elabscience-E-EL-R0012, Elabscience, Houston, TX, USA], IL-6 [Elabscience-E-EL-R0015], IL-10 [Elabscience-E-EL-R0016], TNF-α [Elabscience-E-EL-R2856], and CINC-1 [Thermo Fisher ERCXCL1, Thermo Fisher Scientific, Waltham, MA, USA]) were quantitatively assessed using target-specific, commercially available ELISA kits. Additionally, the supernatant levels of nitric oxide (NO) were quantified as a marker of localized oxidative stress and macrophage activation (Elabscience-E-EL-R0520). All assays were conducted in strict accordance with the respective manufacturers’ validated protocols.

### 2.7. Preparation of Lung Tissue Homogenates and Biochemical Assays

#### 2.7.1. Preparation of Lung Tissue Homogenates

Following lung excision, tissue samples designated for protein analysis were immediately rinsed in ice-cold phosphate-buffered saline (PBS; 0.01 M, pH 7.4, Thermo Fisher Scientific, Waltham, MA, USA) to thoroughly clear residual blood from the pulmonary vasculature. The tissues were subsequently weighed and mechanically minced into small fragments. Minced tissues were homogenized in ice-cold PBS at a standardized 1:9 ratio (tissue weight [g] to PBS volume [mL]) utilizing a pre-chilled glass tissue grinder to prevent thermal degradation of sensitive proteins. The crude homogenate was centrifuged at 5000× *g* for 10 min at 4 °C. The resulting clarified supernatant was carefully aliquoted and preserved for downstream quantification of pro-fibrotic markers and collagen precursors.

#### 2.7.2. Enzyme-Linked Immunosorbent Assays

The concentrations of specific inflammatory biomarkers in the BALF (i.e., IL-1β, IL-6, IL-10, TNF-α, and CINC-1) and critical pro-fibrotic drivers in the lung tissue homogenates (i.e., TGF-β1 [Elabscience-E-EL-0162], WISP-1 [Fine Test-ER0158, Fine Test, Wuhan, China], and TIMP-1 [Elabscience-E-EL-R0540]) were quantitatively assessed using highly specific commercial sandwich ELISA kits. Assays were performed in 96-well microtiter plates pre-coated with target-specific capture antibodies. Briefly, 100 μL of biological samples and serial standard dilutions were added to the designated wells and incubated for 90 min at 37 °C. Following the removal of unbound constituents via a standardized wash buffer, 100 μL of a biotin-conjugated detection antibody working solution was applied, and the plates were incubated for 60 min at 37 °C.

Following three progressive wash cycles, 100 μL of a Streptavidin–Biotin Complex (SABC) working solution was added to amplify the signal, and plates were incubated for an additional 30 min at 37 °C. After a stringent five-cycle wash, 90 μL of 3,3′,5,5′-Tetramethylbenzidine (TMB) chromogenic substrate was introduced, and the plates were incubated in the dark for 10–20 min at 37 °C to allow color development. The enzymatic reaction was arrested by the addition of 50 μL of an acidic stop solution. The optical density (OD) of each well was immediately quantified at a wavelength of 450 nm using a Multiskan FC microplate reader (Thermo Fisher Scientific, Waltham, MA, USA). Target concentrations were extrapolated from highly linear, target-specific standard curves.

#### 2.7.3. Colorimetric Quantification of Hydroxyproline and Nitric Oxide

To evaluate the extent of fibrotic collagen deposition, hydroxyproline (HYP) levels in the lung tissue were quantified using a targeted colorimetric assay (Elabscience-E-EBC-K062-M). Additionally, nitric oxide (NO) levels were measured in the BALF to assess localized oxidative stress.

For the HYP assay, 100 mg of lung tissue (or 100 μL of BALF for NO) was combined with 1 mL of 6 M hydrochloric acid (HCl). The mixture was subjected to robust acid hydrolysis in a 95 °C heat block for 6 h to liberate constituent amino acids. The hydrolysate was subsequently neutralized to a pH of 6.5–7.0. A 2 mL aliquot of the neutralized sample was mixed with 20 mg of a kit-specific clarifying agent and centrifuged at 400× *g* for 10 min. A 400 μL volume of the resulting supernatant (or standard) was mixed with 200 μL of an oxidizing agent and incubated for 15 min at ambient temperature to oxidize the hydroxyproline. Finally, 400 μL of a chromogenic reagent was added, and the samples were incubated in a 60 °C water bath for 15 min to generate the chromophore. The samples were then transferred to a 96-well microplate, and the OD was measured at 558 nm using a microplate reader (Multiskan FC microplate reader, Thermo Fisher Scientific, Waltham, MA, USA). Concentrations were calculated via standard curve regression.

### 2.8. Western Blot Analysis

#### 2.8.1. Protein Extraction and Quantification

Following isolation, approximately 0.5 g of lung tissue from each sample was thoroughly washed with ice-cold PBS to remove residual blood. Tissues were mechanically minced and immediately suspended in ice-cold RIPA lysis buffer (Thermo Fisher Scientific, Waltham, MA, USA), supplemented with a Halt™ Protease and Phosphatase Inhibitor Cocktail containing EDTA, to rigorously prevent proteolytic and autolytic degradation. The tissue suspensions were subjected to sonication to ensure complete cellular disruption and shearing of genomic DNA and subsequently incubated at 4 °C for 2 h with gentle agitation to maximize protein solubilization. The crude lysates were then clarified by centrifugation at 12,000× *g* for 10 min at 4 °C. The protein-enriched supernatant was carefully aliquoted, and the total protein concentration was precisely determined utilizing the Bradford protein assay via spectrophotometry.

#### 2.8.2. SDS-PAGE and Membrane Transfer

Lysate aliquots containing exactly 60 μg of total protein were prepared for electrophoresis by combining the extract with a 4× Laemmli sample loading buffer supplemented with the reducing agent β-mercaptoethanol. The samples were thermally denatured at 95 °C for 5 min. The prepared protein suspensions (30 μL per well) were then resolved via 10% sodium dodecyl sulfate–polyacrylamide gel electrophoresis (SDS-PAGE). The electrophoretic separation was conducted at 60 V through the stacking gel to concentrate the samples, followed by 140 V through the resolving gel for approximately one hour until the tracking dye reached the distal boundary of the gel (mPAGE Mini Gel Tank, Merck, Darmstadt, Germany). Following resolution, the fractionated proteins were electroblotted onto a solid-phase transfer membrane utilizing a rapid dry-transfer blotting system for 7 min.

#### 2.8.3. Immunoblotting and Fluorescent Detection

To abrogate non-specific antibody binding, the post-transfer membranes were incubated in LI-COR Blocking Buffer for 1 h at ambient temperature. The membranes were subsequently incubated overnight at 4 °C under constant, gentle agitation with target-specific primary antibodies directed against α-SMA (1:500 dilution) and MMP-12 (1:1000 dilution, Thermo Fisher Scientific, Waltham, MA, USA). A primary antibody targeting the housekeeping protein GAPDH was included in the multiplexed solution to serve as the endogenous loading control. All primary antibodies were formulated in LI-COR Antibody Diluent.

Following three 10 min wash cycles in Tris-buffered saline containing 0.1% Tween-20 (TBST), the membranes were probed with species-appropriate, near-infrared fluorophore-conjugated secondary antibodies for 45 min at ambient temperature, strictly protected from light. After a final series of three 10 min TBST washes to thoroughly clear unbound secondary conjugates, the specific protein bands were visualized, and their fluorescent signals were digitally acquired using the LI-COR Odyssey M Infrared Imaging System (LI-COR Biosciences, Lincoln, NE, USA).

### 2.9. Tissue Harvesting and Gross Pathological Examination

#### 2.9.1. Vascular Perfusion and Macroscopic Assessment

Following euthanasia, a median sternotomy was performed to fully expose the thoracic cavity. To thoroughly clear the pulmonary vasculature of circulating erythrocytes, which can interfere with downstream colorimetric assays and histological imaging, the lungs were immediately perfused with ice-cold PBS. Following perfusion and prior to any structural dissection, the intact lungs were subjected to a comprehensive macroscopic evaluation. The tissue was carefully inspected for gross pathological abnormalities, including focal discoloration (e.g., hemorrhagic, necrotic, or fibrotic lesions), textural alterations (e.g., edematous bogginess or fibrotic induration), and abnormal morphometric variations in lobe size or volume (Table S1).

#### 2.9.2. Tissue Allocation and Fixation

Following the gross anatomical evaluation, the lungs were strategically partitioned to support diverse downstream analytical techniques. The right main bronchus was securely ligated utilizing a surgical suture, and the multi-lobed right lung was sharply excised. These right lobes were immediately preserved and allocated for subsequent tissue homogenization, biochemical assays, Western blot analyses, and qRT-PCR evaluations. Conversely, to optimally preserve the delicate alveolar architecture for morphological assessment, the single-lobed left lung was subjected to structural fixation. The left lung was gently inflated via the steady i.t. instillation of 10% neutral buffered formalin (NBF, Oxford Lab Fine Chem, Mumbai, India) to prevent alveolar collapse. The fully inflated left lobe was then completely submerged in a 10% NBF bath for continuous fixation prior to downstream histopathological and immunohistochemical processing.

### 2.10. Histopathological Examination of Lung Tissue

#### 2.10.1. Tissue Processing and Microtomy

The formalin-fixed left lung lobes were utilized for all downstream histopathological evaluations. Following adequate fixation, the tissues were processed using standard automated tissue preparation protocols, which included dehydration through an ascending graded ethanol series, clearing in xylene, and final embedding in paraffin wax. Utilizing a rotary microtome, the paraffin-embedded tissue blocks were sectioned at a uniform thickness of 5 µm. The resulting sections were carefully floated onto a heated water bath and mounted on precisely labeled glass slides.

#### 2.10.2. Hematoxylin and Eosin Staining

To evaluate the general pulmonary architecture, the mounted tissue sections were subjected to a standardized, manual Hematoxylin and Eosin (H&E) staining protocol. The sections were first deparaffinized through successive xylene baths and subsequently rehydrated through a descending graded alcohol series down to distilled water. The hydrated tissue sections were then stained with hematoxylin to highlight nuclear morphology. Following a brief differentiation step in acid alcohol to remove non-specific background staining, the sections were blued in a dilute aqueous ammonia solution. The slides were subsequently counterstained with eosin to visualize cytoplasmic and ECM components. Finally, the stained sections were dehydrated through an ascending graded isopropyl alcohol series, cleared in two terminal xylene baths to prepare the tissue for optical clarity, and permanently coverslipped using DPX mounting medium (Sigma-Aldrich, Darmstadt, Germany).

#### 2.10.3. Picrosirius Red Staining

To visualize collagen networks, the slides were incubated in a Picrosirius Red solution (0.1% *w/v* Sirius Red F3B in saturated aqueous picric acid) for 60 min. Following incubation, the slides were washed in two changes of acidified water (0.5% glacial acetic acid) for 2 min each to prevent the loss of the Sirius Red dye. The sections were then vigorously shaken to remove excess water, rapidly dehydrated through three changes of 100% ethanol, cleared in two changes of xylene, and coverslipped using a resinous mounting medium.

Histological evaluation was performed using both brightfield and polarized light microscopy. Total collagen deposition was assessed under brightfield illumination, where collagen fibers appeared red against a yellow cytoplasmic background. Polarized light microscopy was utilized to differentiate collagen fiber thickness based on birefringence, distinguishing thicker fibers (predominantly Type I, displaying orange-red/yellow birefringence) from thinner fibers (predominantly Type III, displaying pale green birefringence).

#### 2.10.4. Microscopic Evaluation and Fibrosis Scoring

The histological slides were de-identified, assigned randomized alphanumeric codes, and evaluated by independent investigators who remained strictly blinded to both the treatment allocation and the specific experimental lung injury model. The fully cured slides were examined using a bright-field light microscope. The tissue sections were systematically evaluated to assess the extent of structural disruption and acute inflammation. Specifically, the qualitative evaluation focused on alveolar septal thickening, the presence of alveolar and vascular congestion, and the degree of perivascular and peribronchial leukocytic infiltration.

To provide a robust, quantitative assessment of the fibrotic remodeling and collagen deposition, the severity of the observed histopathological abnormalities was graded using the standardized Ashcroft scoring system (Table S2). This well-established histological scale was utilized to objectively categorize the extent of fibrotic progression and architectural distortion across the varied LI models. To further evaluate the quality of the collagen present in the lungs, grading of the Picrosirius-Red-stained slides was performed. Bright yellow collagen type I fibers served as an indicator of a healthy state or mild LI (Table S3).

### 2.11. Immunohistochemistry

#### 2.11.1. Tissue Preparation and Antigen Retrieval

Immunohistochemical evaluations were conducted on the 10% NBF-fixed, paraffin-embedded left lung tissue specimens. Tissue blocks were sectioned at a uniform thickness of 4 μm and mounted onto positively charged, poly-L-lysine-coated glass slides to ensure robust tissue adhesion during rigorous procedural washing. The sections were systematically deparaffinized through successive xylene baths and rehydrated through a descending graded alcohol series to distilled water. To unmask structural epitopes cross-linked during formalin fixation, the sections were subjected to heat-induced epitope retrieval (HIER). This was achieved by immersing the slides in an appropriate retrieval buffer (citrate- or EDTA-based, dependent on specific antibody optimization) within a pressurized decloaking chamber. Following the pressurized heating cycle, the chamber was gradually depressurized and allowed to cool to ambient temperature to facilitate proper epitope refolding.

#### 2.11.2. Immunolabeling and Chromogenic Detection

Following antigen retrieval, the slides were thoroughly rinsed in Tris-buffered saline (TBS, pH 7.4). To prevent non-specific background staining, endogenous tissue peroxidase activity was effectively quenched by incubating the sections with a dedicated Peroxidase Suppressor (Thermo Fisher Scientific, Waltham, MA, USA) for 30 min. Following this pre-treatment, the sections were incubated with the specific primary antibodies.

After rigorous washing in TBS to remove unbound primary complexes, the tissues were incubated for 30 min at ambient temperature with a ready-to-use, horseradish peroxidase (HRP)-conjugated secondary polymer system (Dako REAL™ EnVision™ Rabbit/Mouse HRP; Dako, Glostrup, Denmark). Target visualization and chromogenic signal detection were achieved through the targeted application of 3,3′-diaminobenzidine (DAB), which precipitates as a distinct brown stain at the sites of antigen localization. Finally, the sections were lightly counterstained with Mayer’s hematoxylin to provide nuclear contrast, dehydrated through an ascending graded alcohol series, cleared in xylene, and permanently mounted with a resinous medium for high-resolution microscopic evaluation.

### 2.12. Quantitative Reverse Transcription PCR

#### 2.12.1. Tissue Preservation and RNA Isolation

Immediately following euthanasia and PBS perfusion, approximately 0.5 g of the ligated right lung tissue was mechanically minced and rapidly submerged in RNAlater™ Stabilization Solution (Thermo Fisher Scientific, Waltham, MA, USA) to prevent RNA degradation. The samples were incubated for 2 h at 2–4 °C to allow adequate tissue penetration of the stabilizer, and subsequently frozen at −20 °C.

For total RNA extraction, the stabilized tissues were subjected to cellular disruption using the TaqMan™ Fast Advanced Cells-to-CT™ Kit lysis buffer system (Thermo Fisher Scientific, Waltham, MA, USA). Briefly, a working lysis solution was prepared by adding 5 µL of DNase I to 495 µL of Lysis Buffer. A 1.5 mL volume of this active lysis solution was added to each minced tissue sample, mixed thoroughly by vortexing, and incubated at ambient temperature for 5 min to facilitate cellular membrane lysis and genomic DNA digestion. The reaction was arrested by the addition of 15 µL of Stop Solution, followed by a 2 min ambient incubation. Total RNA was subsequently purified from the resulting crude lysate using a commercial RNA isolation kit (New England Biolabs, Ipswich, MA, USA) in strict accordance with the manufacturer’s spin-column protocol.

#### 2.12.2. RNA Quantification and cDNA Synthesis

The concentration and purity of the eluted total RNA were spectrophotometrically evaluated using a NanoDrop™ microvolume spectrophotometer (Thermo Fisher Scientific, Waltham, MA, USA). RNA concentration was recorded in ng/µL (measuring absorbance at 260 nm), and sample purity was validated via the A_260_/A_280_ absorbance ratio (with an acceptable high-purity threshold of ~2.0).

Reverse transcription (RT) was performed to synthesize complementary DNA (cDNA). A master mix was formulated combining 25 µL of 2× Fast Advanced RT Buffer, 2.5 µL of Fast Advanced RT Enzyme Mix, and 12.5 µL of nuclease-free water. For each reaction, 10 µL of the purified RNA sample was added to 40 µL of the RT master mix. The RT thermal profile was executed in a thermal cycler, consisting of an initial transcription step at 37 °C for 30 min, followed by an enzyme inactivation step at 95 °C for 5 min. The resulting cDNA was stored at −20 °C until amplification.

#### 2.12.3. Quantitative Real-Time PCR and Data Analysis

Gene expression profiling was conducted using a fluorogenic probe-based qPCR assay. Target-specific primers and specific probes (BioServe Biotechnologies, Hyderabad, India) were reconstituted in nuclease-free water. An intermediate 20× working stock solution was prepared, containing 18 µM of both forward and reverse primers and 5 µM of the target probe.

The qPCR reactions were carried out in a final volume of 10 µL per well, prepared by distributing 6 µL of the qPCR master mix cocktail (including the 20× primer/probe stock) and 4 µL of the cDNA template into a 384-well microplate (PerkinElmer, Waltham, MA, USA) or a MicroAmp™ Fast Optical 96-Well Reaction Plate (Applied Biosystems, Waltham, MA, USA). The thermal cycling conditions were programmed as follows: Uracil-DNA Glycosylase (UDG) activation at 50 °C for 2 min, polymerase activation at 95 °C for 2 min, followed by 40 cycles of denaturation at 95 °C for 3 s, and annealing/extension at 60 °C for 20 s.

Relative mRNA expression levels were calculated based on the comparative threshold cycle (C_t_) method, utilizing the 2^–ΔΔCt^ equation (QuantStudio 6 Flex (v1.7.2), Applied Biosystems, Waltham, MA, USA). Data were normalized to the expression of the endogenous *GAPDH* housekeeping gene. The specific primer sequences utilized for the targeted transcripts are listed in Table S4.

### 2.13. Bias Mitigation and Blinding

To rigorously mitigate bias throughout the study duration, strict blinding protocols were enforced. Following terminal sacrifice, all harvested tissues and BALF samples were de-identified and assigned randomized alphanumeric codes. Beyond the independent, blinded histological evaluation, all downstream biochemical quantifications, including ELISA, hydroxyproline colorimetric assessments, Western blot analyses, and qRT-PCR, were executed by technical operators who remained blinded to the sample identities and group allocations until the final data acquisition and statistical compilation phases.

### 2.14. Statistical Analysis

#### 2.14.1. Data Processing and Statistical Comparisons

All quantitative experimental data are expressed as the mean ± standard deviation (SD) or standard error of the mean (SEM). Prior to parametric testing, the data were rigorously evaluated for normal distribution (Shapiro–Wilk test) and homogeneity of variances (Levene’s test).

For the direct statistical comparison of continuous variables between two independent experimental groups, an unpaired Student’s *t*-test was applied. For multiple group comparisons (specifically assessing differences across the sham control, disease (vehicle) control, and the three LFCM dose cohorts), a one-way analysis of variance (ANOVA) was utilized. Following a statistically significant ANOVA result, Dunnett’s post hoc test was applied to specifically compare the mean of each LFCM therapeutic group directly against the mean of the positive (disease) control group.

#### 2.14.2. Software and Significance Thresholds

All statistical computations and dataset evaluations were performed using the Minitab Statistical Software, Version 21.3.1 (Minitab, State College, PA, USA). The threshold for statistical significance was predefined at an alpha level of 5% (*p* < 0.05). Graphical presentations of the statistical data and final figure assemblies were generated utilizing Microsoft Excel and PowerPoint (Microsoft Corporation, Redmond, WA, USA).

## 3. Results

### 3.1. Clinical Observations and Hematological Profiling

#### 3.1.1. Survival, Morbidity, and Body Weight Dynamics

**Figure 1:**
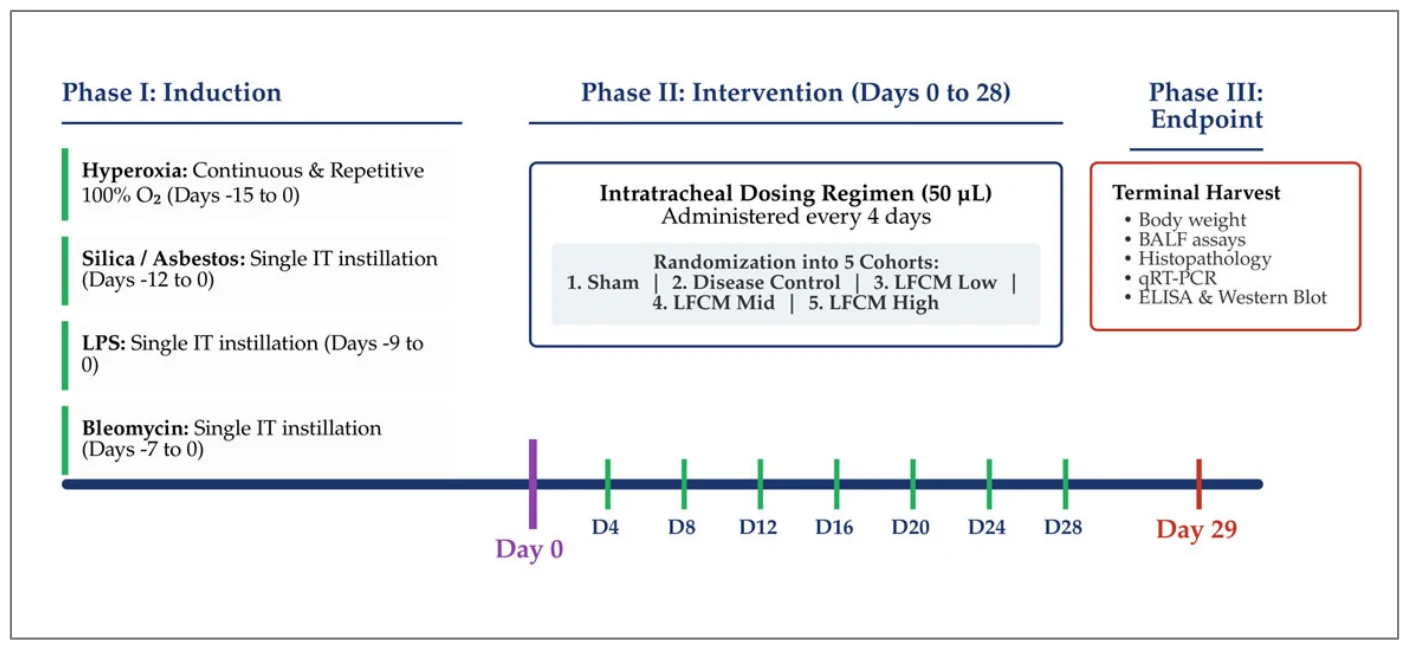
Schematic representation of the experimental design and interventional timeline across the five study arms. Phase I (Induction) outlines the specific temporal windows required to establish baseline disease pathology prior to treatment. Induction regimens included continuous and repetitive exposure to 100% O_2_ for hyperoxia; a single i.t. instillation of silica or asbestos with a 10 to 12-day developmental window; a single i.t. instillation of LPS for LI developing over 7 to 9 days; and a single i.t. instillation of bleomycin for LI developing over 5 to 7 days. Phase II (Intervention) spanned day 0 to day 28, marking the therapeutic window where symptomatic rats were randomized into five groups. Animals received a 50 µL i.t. administration of either a vehicle control or the respective LFCM dose every 4 days. Phase III (Endpoint) denotes the terminal harvest on day 29 for downstream macroscopic and molecular evaluations. LFCM, lung fibroblast conditioned medium; LPS, lipopolysaccharides.

Throughout the entire 28-day therapeutic intervention period, 100% survival was maintained across all five study arms (hyperoxia, LPS, asbestos, silica, and bleomycin LI models). Furthermore, no overt signs of severe morbidity or immediate procedural toxicity were observed in any of the cohorts following the repeated i.t. administrations (Figure 1).

Body mass trajectories, however, exhibited disease model- and dose-dependent fluctuations over the study duration. When compared to the vehicle-treated disease cohorts (G2-Positive Control), statistically significant transient reductions in animal body weight were noted in specific LFCM treatment groups. In the hyperoxia arm, significant decreases were recorded for the G4-Mid dose on day 24, and the G5-High dose on days 24, 28, and 29. Similarly, the LPS arm exhibited significant body weight reductions in the G5-High dose group by days 28 and 29. The asbestos arm demonstrated the most frequent fluctuations, with significant weight reductions observed in the G3-Low dose and G5-High dose groups across multiple time points (days 16, 20, 24, 28, and 29), as well as in the G4-Mid dose group on day 20. In the bleomycin arm, an early but isolated weight reduction was noted in the G5-High dose group on day 8. Conversely, animals subjected to the silica-induced lung injury model demonstrated stable weight trajectories, with no significant treatment-associated body weight alterations observed across any LFCM dosage tier.

#### 3.1.2. Localized Pulmonary Cellularity (BALF Profile)

**Figure 2:**
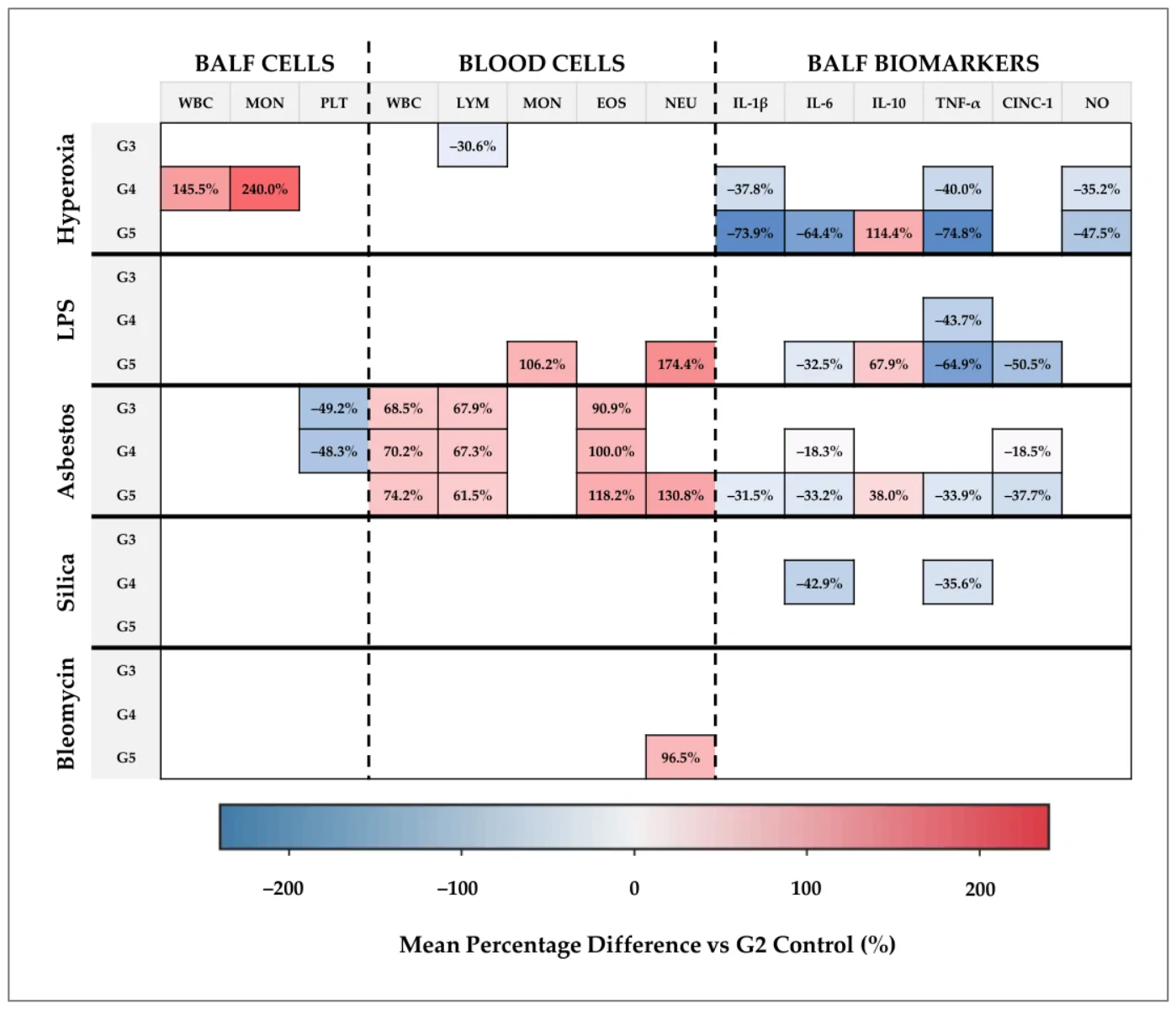
Quantitative modulation (i.e., statistically significant, *p* < 0.05) of localized and systemic cellular profiles and alveolar inflammatory biomarkers following FE002-Lu LFCM treatment. The heatmap visualizes the relative changes in cell populations within the BALF and peripheral blood, alongside specific pro- and anti-inflammatory mediator concentrations in the BALF. Color intensities represent the modulation of the LFCM-treated cohorts (G3-Low, G4-Mid, and G5-High doses) compared directly against their respective vehicle-treated disease control groups (G2-Control) across the five study arms (hyperoxia, LPS, asbestos, silica, and bleomycin). Red hues indicate an upregulation of the respective cell type/biomarker, whereas blue hues indicate downregulation. LFCM, lung fibroblast conditioned medium; BALF, bronchoalveolar lavage fluid.

Analysis of the BALF revealed distinct, model-specific shifts in localized alveolar cellularity following LFCM treatment. In the hyperoxia arm, LFCM administration resulted in a localized cellular influx, evidenced by a statistically significant increase in total white blood cell (WBC) and monocyte (MON) counts within the BALF of the G4-Mid dose group (Figure 2, Table S5).

Interestingly, LFCM treatment appeared to exert a suppressive effect on local alveolar thrombosis and platelet pooling. Noticeable reductions in BALF platelet counts were a consistent feature across multiple lung injury etiologies, manifesting almost significantly in the G5-High dose groups of the hyperoxia, LPS, and bleomycin arms. In the asbestos arm, a significant reduction in BALF platelets occurred at the lower therapeutic tiers (G3-Low and G4-Mid dose groups). Mirroring the clinical observation data, the silica arm exhibited no significant treatment-related variations in local BALF cell count parameters across any of the evaluated LFCM dose groups (Figure 2).

#### 3.1.3. Systemic Hematological Responses (Peripheral Blood Profile)

Evaluation of the peripheral whole blood profiles revealed that localized i.t. LFCM administration also modulated systemic leukocytic responses. In the hyperoxia arm, systemic analysis indicated a significant decrease in circulating lymphocyte counts specifically within the G3-Low dose group. Conversely, the LPS model, representing an acute infectious trigger, demonstrated a significant systemic upregulation of innate immune cells, marked by elevated blood neutrophil and monocyte counts in the G5-High dose cohort (Figure 2).

The asbestos model triggered the broadest systemic hematological activation among the evaluated lung injury pathways. Compared to positive controls, LFCM treatment in asbestos-injured rats resulted in significant systemic increases in total WBC, lymphocyte, and eosinophil counts across all therapeutic tiers (G3-Low, G4-Mid, and G5-High), alongside an isolated significant elevation in circulating neutrophils within the G5-High dose group. For the bleomycin-induced lung fibrosis model, the G5-High dose group exhibited a significant increase in systemic neutrophil counts, coupled with a noticeable elevation in peripheral platelet counts in both the G4-Mid and G5-High dose groups. Consistent with both body weight and BALF metrics, the silica arm demonstrated no significant treatment-associated alterations in the systemic hematological profile (Figure 2).

### 3.2. Pulmonary Cytokine Dynamics and Inflammatory Modulation in BALF

#### 3.2.1. Suppression of Oxidative and Infectious Cytokine Storms

Therapeutic intervention with LFCM exerted a profound modulatory effect on the localized alveolar cytokine storm, effectively blunting acute inflammatory cascades while promoting resolution pathways. In the hyperoxia-induced oxidative lung injury model, treatment significantly attenuated pro-inflammatory signaling. Specifically, significant decreases in IL-1β, TNF-α, and localized nitric oxide (NO) levels were observed in both the G4-Mid and G5-High dose groups compared to positive controls (Figure 2).

Furthermore, the hyperoxia G5-High dose group exhibited a significant reduction in IL-6, which was uniquely coupled with a significant elevation in the potent anti-inflammatory cytokine IL-10, indicating a phenotypic shift toward tissue damage resolution. Similar immunomodulatory efficacy was observed in the LPS-induced infectious model. The LPS G5-High dose group demonstrated a comprehensive suppression of the acute phase response, evidenced by significant decreases in IL-6, TNF-α, and the neutrophil chemoattractant CINC-1. Concurrently, this high-dose cohort exhibited a significant, compensatory increase in IL-10 levels. The G4-Mid dose also demonstrated targeted anti-inflammatory activity, characterized by a specific and significant reduction in TNF-α levels (Figure 2).

#### 3.2.2. Modulation of Particulate-Induced Inflammation

In the occupational particulate models, LFCM successfully mitigated the chronic macrophage-driven inflammatory response. In the asbestos-induced lung injury model, the G5-High dose group demonstrated significant decreases in the primary pro-inflammatory cytokines IL-1β and TNF-α. Furthermore, significant dose-dependent reductions in IL-6 and CINC-1 levels were observed across both the G4-Mid and G5-High dose groups. Consistent with the oxidative and infectious models, this robust suppression of pro-inflammatory mediators in the asbestos G5-High group was paired with a statistically significant elevation in tissue-resolving IL-10 levels (Figure 2).

In the silica-induced lung injury model, LFCM exhibited a narrower but defined therapeutic window. Significant decreases in crucial pro-inflammatory drivers, specifically IL-6 and TNF-α, were effectively isolated to the G4-Mid dose group, suggesting an optimal localized dosing threshold for silica-induced alveolar inflammation (Figure 2).

#### 3.2.3. Cytokine Trends in Drug-Induced Fibrosis

In the bleomycin-induced pulmonary fibrosis model, the therapeutic application of LFCM resulted in consistent, dose-dependent biological trends; however, these localized cytokine shifts did not reach statistical significance within the 28-day evaluation window. While the data demonstrated a broad pattern of suppression across pro-inflammatory markers (IL-1β, IL-6, TNF-α, and CINC-1) coupled with a concomitant elevation in the anti-inflammatory cytokine IL-10 within the G4-Mid and G5-High dose groups, these shifts must be interpreted cautiously. The lack of statistical significance suggests that a 28-day observation period may be insufficient to capture the complete immunomodulatory inflection point in this delayed, drug-induced fibrosis model. Nevertheless, these observed trends suggest an active, ongoing modulation of the profibrotic microenvironment that functionally aligns with the statistically significant downstream structural and pre-translational anti-fibrotic improvements achieved in these same cohorts.

### 3.3. Modulation of Pro-Fibrotic Drivers and ECM Remodeling

#### 3.3.1. Attenuation of Fibrogenic Mediators and Collagen Deposition

**Figure 3:**
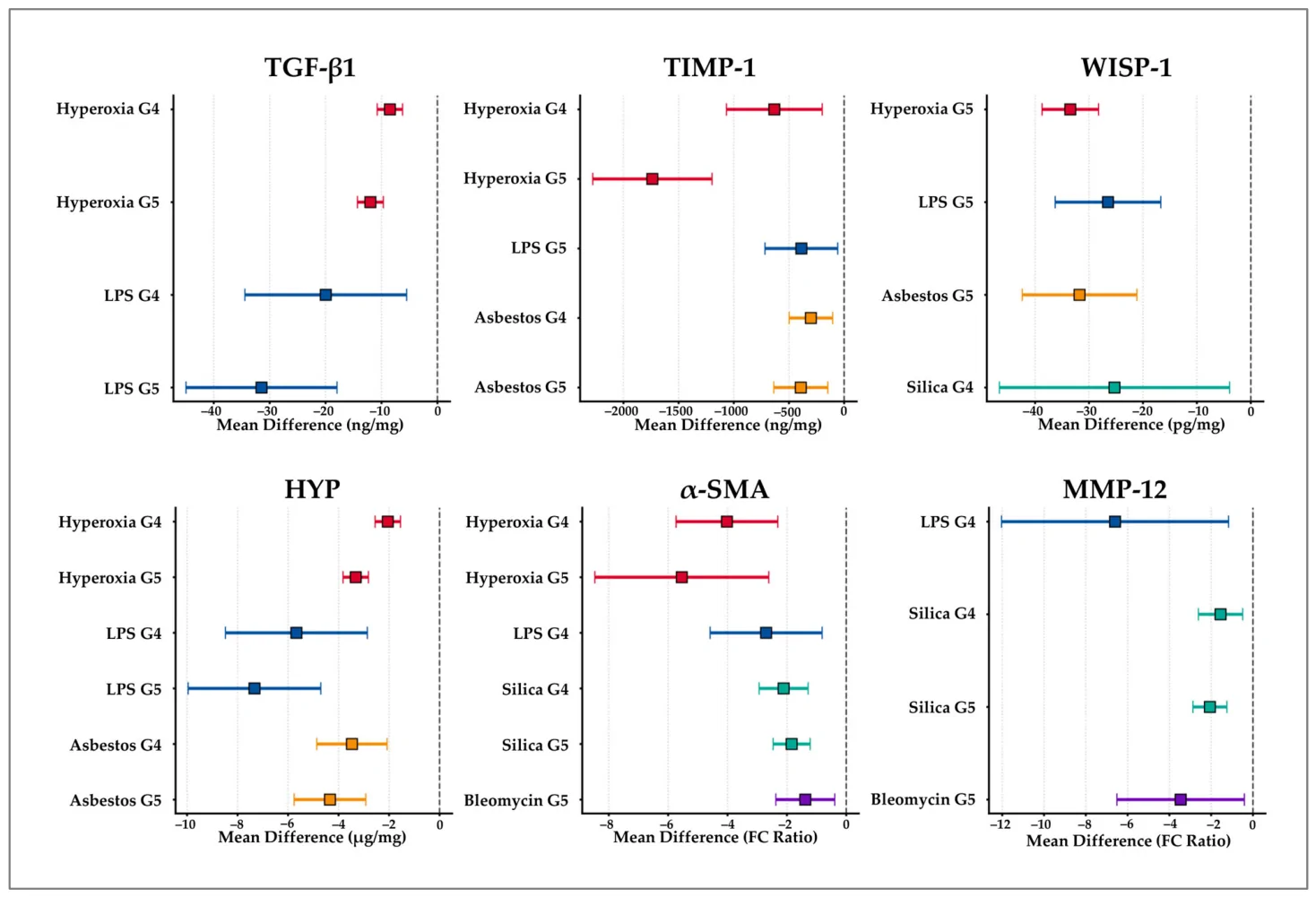
Quantitative modulation (i.e., statistically significant, *p* < 0.05) of pro-fibrotic mediators, ECM components, and tissue remodeling effectors in lung tissue homogenates. The multi-panel forest plot visualizes alterations in the concentrations of key fibrogenic biomarkers (TGF-β1, TIMP-1, WISP-1) and collagen precursors (hydroxyproline, HYP), alongside the relative protein expression fold-change (FC ratio) of structural and remodeling effectors (α-SMA and MMP-12). Protein expression of the structural and remodeling effectors was assessed via Western blot. Data points represent the calculated mean difference between the LFCM-treated cohorts and the mean baseline values of their respective vehicle-treated disease control groups (G2-Control) across the five study arms. Error bars denote the 95% confidence intervals, derived from the standard deviations (± SD). The vertical dashed line at zero indicates no treatment effect (treatment equals disease control); data points falling to the left of this line indicate a downregulation relative to the disease control. Absolute concentrations for the tissue homogenate biomarkers are standardized to total protein and specified as ng/mg, pg/mg, or µg/mg. LFCM, lung fibroblast conditioned medium; HYP, hydroxyproline; FC, fold-change.

Quantitative biochemical evaluations of lung tissue homogenates demonstrated that FE002-Lu LFCM treatment effectively blunted the molecular drivers of fibrotic progression and pathological ECM deposition. In both the acute oxidative (hyperoxia) and infectious (LPS) injury models, the G5-High dose groups exhibited a comprehensive and statistically significant downregulation of critical pro-fibrotic targets, including the canonical fibrogenic cytokine TGF-β1, the profibrotic matricellular protein WISP-1, the matrix-stabilizing TIMP-1, and hydroxyproline (HYP, a primary biochemical surrogate for total collagen content), relative to vehicle-treated positive controls (Figure 3, Table S6).

This potent anti-fibrotic effect was also evident at lower therapeutic thresholds; the G4-Mid dose significantly reduced TGF-β1, TIMP-1, and HYP levels in the hyperoxia model, and successfully attenuated TGF-β1 and HYP in the LPS model (Figure 3). In the particulate-induced lung injury models, LFCM treatment similarly constrained aberrant fibrotic remodeling. The asbestos model demonstrated significant decreases in TIMP-1 and HYP levels across both the G4-Mid and G5-High dose groups, coupled with a significant reduction in WISP-1 expression that was exclusive to the G5-High dose cohort. In the silica-induced lung injury model, targeted anti-fibrotic activity was observed, with a significant reduction in WISP-1 specifically localized to the G4-Mid dose group. Taken together, these data are indicative of an active, dose-dependent suppression of the fibrotic cascade (Figure 3).

#### 3.3.2. Inhibition of Myofibroblast Activation and Tissue Remodeling Effectors

Western blot analyses corroborated the tissue homogenate findings, demonstrating potent, cellular-level inhibition of key structural effectors involved in irreversible lung remodeling. Crucially, LFCM administration successfully suppressed myofibroblast transdifferentiation. This was evidenced by significant decreases in the protein expression of α-SMA, the definitive marker of activated, collagen-secreting myofibroblasts, within the hyperoxia (G4-Mid and G5-High), LPS (G4-Mid), silica (G4-Mid and G5-High), and bleomycin (G5-High) cohorts (Figure 3).

Furthermore, targeted modulations in pathological ECM turnover mechanisms were recorded. Significant decreases in the protein expression of macrophage metalloelastase (MMP-12), an enzyme heavily implicated in destructive matrix remodeling and chronic inflammation, were distinctly observed in the LPS (G4-Mid), silica (G4-Mid and G5-High), and bleomycin (G5-High) dose groups (Figure 3).

### 3.4. Histopathology, Tissue Architecture, and Gross Pathology

#### 3.4.1. Macroscopic Evaluation and Gross Pathology

Macroscopic and gross pathological evaluations demonstrated that LFCM intervention successfully shifted the pulmonary phenotype from a state of chronic, irreversible structural degradation to milder, resolving tissue states. In the LPS cohort, gross active fibrogenesis was completely absent in the G5-High dose group, in stark contrast to the widespread fibrotic remodeling observed in the vehicle-treated positive controls.

In the progressive asbestos and bleomycin models, the vehicle control groups exhibited severe, end-stage pathology characterized by dense collagenous induration and chronic, hemosiderin-laden inflammation. Conversely, the high-dose LFCM treatments effectively halted this progression; the G5-High dose groups in both the asbestos and bleomycin models presented predominantly with acute focal hemosiderin accumulation, entirely lacking the dense fibrotic consolidation seen in the controls. Similarly, within the silica arm, half of the G5-High dose cohort exhibited localized signs limited to acute alveolitis, whereas the corresponding positive controls demonstrated advanced chronic inflammation and widespread structural damage.

#### 3.4.2. Histopathological Assessment and Fibrosis Scoring

**Figure 4:**
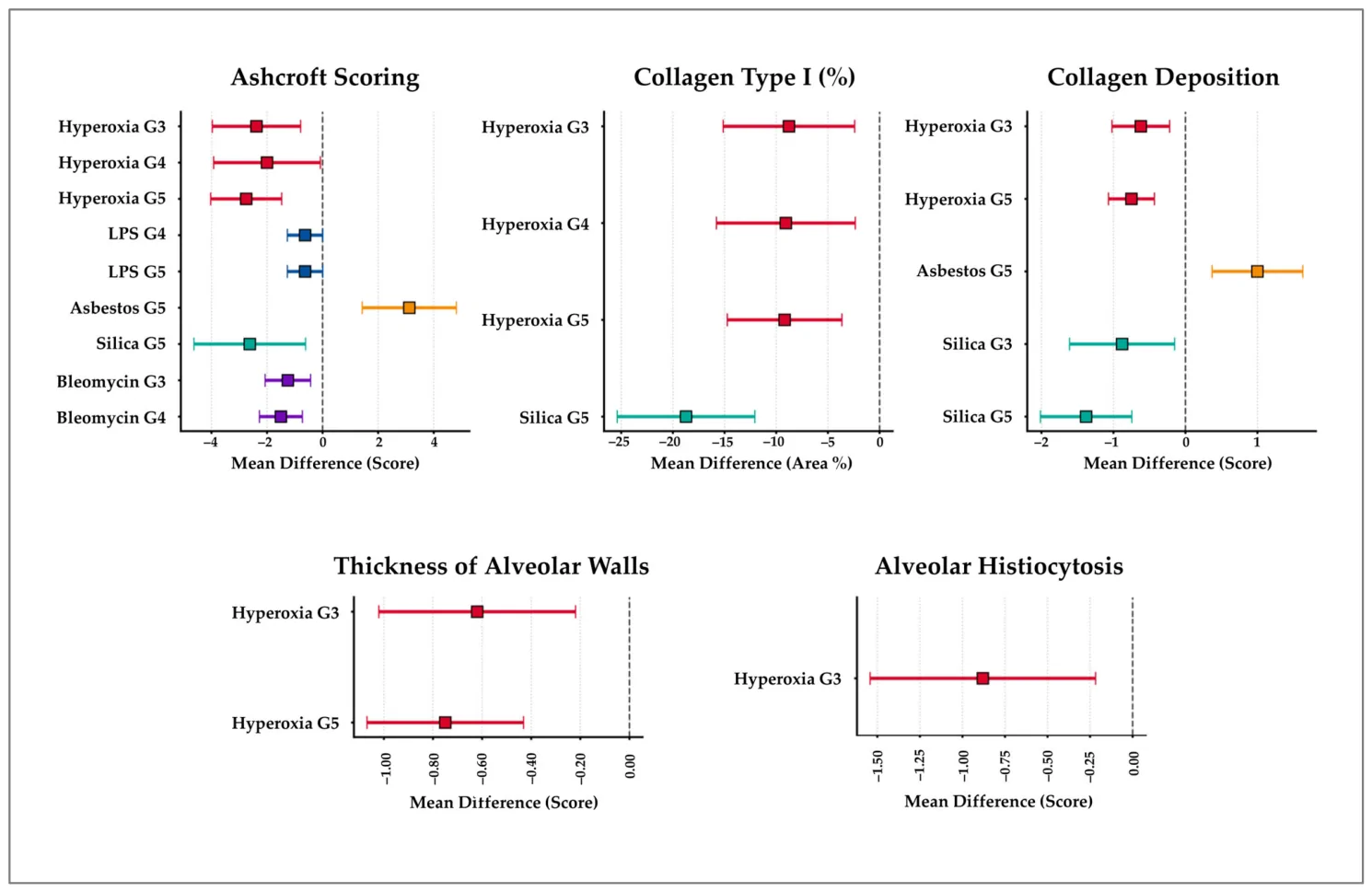
Quantitative histopathological assessment, structural preservation, and collagen deposition profiles across LI models following LFCM treatment. The multi-panel forest plot visualizes statistically significant (*p* < 0.05) alterations in pulmonary microarchitecture, structural degradation, and fibrotic tissue distortion following therapy. Evaluated parameters across the panels include the standardized Ashcroft fibrosis score, the total area percentage of collagen type I, localized collagen deposition scoring, morphometric thickness of alveolar walls, and alveolar histiocytosis severity. Data points represent the calculated mean difference between the LFCM-treated therapeutic cohorts (G3-Low, G4-Mid, and G5-High doses) and the mean baseline values of their respective vehicle-treated disease control groups (G2-Control) across the five study arms. Error bars denote the 95% confidence intervals derived from the measured SD. The vertical dashed line at zero signifies no treatment effect (treatment equals disease control). Data points positioned to the left of this line denote a reduction (downregulation) in pathological severity or collagen deposition relative to the disease baseline. Specific parameters are quantified as either area percentages (for collagen type I) or semi-quantitative abnormality and severity scores (for all other structural metrics). LFCM, lung fibroblast conditioned medium.

Microscopic preservation of the delicate alveolar architecture was quantitatively validated utilizing the standardized Ashcroft scoring system. LFCM treatment significantly attenuated fibrotic tissue distortion across the majority of the lung injury etiologies. Statistically significant reductions in Ashcroft scores were recorded in the hyperoxia (G3-Low, G4-Mid, and G5-High), LPS (G4-Mid and G5-High), silica (G5-High), and bleomycin (G3-Low and G4-Mid) treatment groups relative to their respective positive controls (Figure 4, Table S7).

Corroborating these architectural improvements, significant quantitative decreases in the overall percentage of collagen type I deposition were confirmed in the hyperoxia (G3-Low, G4-Mid, and G5-High) and silica (G5-High) dose groups (Figure 4).

Notably, a divergent, anomalous response was observed in the asbestos-induced lung injury model. In this specific cohort, the G5-High dose group exhibited a paradoxical, statistically significant increase in both Ashcroft scores and total collagen deposition compared to the positive control group. This suggests a potential localized hypersensitivity or dose-limiting localized toxicity at the highest LFCM concentration when interacting directly with highly retained asbestos fibers, highlighting the need for precise dose optimization in certain particulate exposures (Figure 4).

#### 3.4.3. Visualization of Collagen Fibril Architecture

To further evaluate pulmonary ECM resilience and collagen fibril organization, tissue sections were subjected to Picrosirius Red staining and visualized under polarized light. In this assay, the presentation of thin, bright yellow birefringent fibers is indicative of loosely packed, newly remodeling collagen associated with mild or resolving LI, contrasting sharply with the dense, thick red-orange birefringence characteristic of mature, heavily cross-linked fibrotic scars.

A markedly higher prevalence of these resolving mild-injury indicators (yellow fibers) was observed in the LFCM-treated groups compared to their respective positive controls across all five study arms. Specifically, an increased incidence of resilient tissue architecture was noted in the hyperoxia (G4: *n* = 5, G5: *n* = 6 vs. Control: *n* = 3), LPS (G4: *n* = 7, G5: *n* = 4 vs. Control: *n* = 2), asbestos (G4: *n* = 4, G5: *n* = 2 vs. Control: *n* = 3), silica (G5: *n* = 4 vs. Control: *n* = 2), and bleomycin (G4: *n* = 5, G5: *n* = 4 vs. Control: *n* = 3) cohorts, visually confirming LFCM’s capacity to disrupt pathological collagen cross-linking and preserve functional lung parenchyma (Figure S1).

### 3.5. Immunohistochemistry and Transcriptional Profiling

#### 3.5.1. Immunohistochemical Localization of Lysosomal and Proteolytic Enzymes

Immunohistochemical (IHC) analyses revealed complex, etiology-dependent modulations of lysosomal activity and proteolytic matrix turnover within the pulmonary microenvironment. In the models characterized by acute, aggressive inflammation (hyperoxia and LPS), LFCM treatment appeared to effectively stabilize lysosomal and macrophage hyperactivation. This stabilization was evidenced by almost significant, dose-dependent reductions in both Cathepsin-D and MMP-12 H-scores across all therapeutic tiers (G3-Low, G4-Mid, and G5-High).

**Figure 5:**
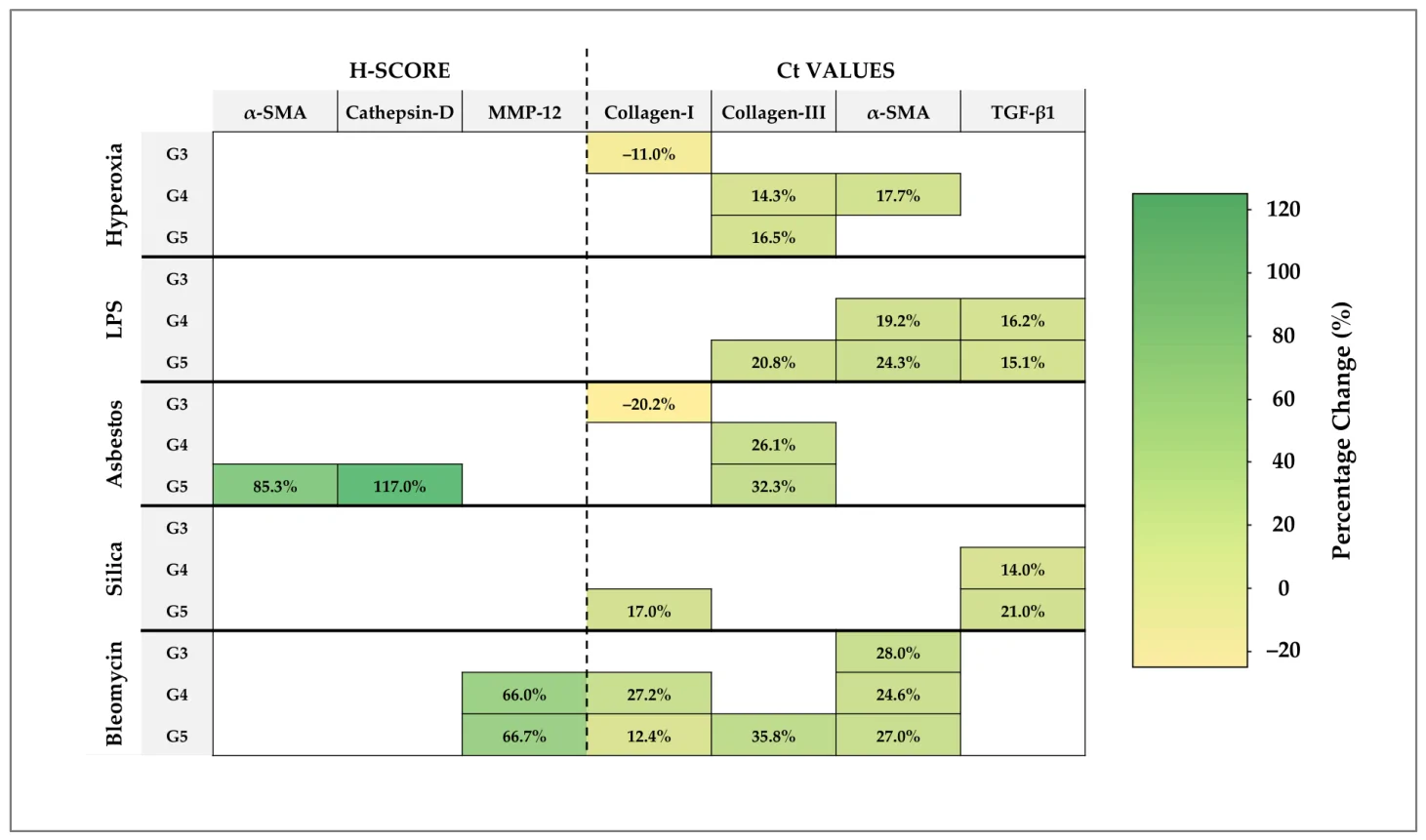
Parallel IHC and transcriptional profiling of tissue remodeling effectors and fibrillar collagens following LFCM treatment. The heatmap visualizes statistically significant (*p* < 0.05) alterations in localized protein accumulation and pre-translational mRNA expression levels across the five study arms. Color intensities represent the relative change in the LFCM therapeutic cohorts (G3-Low, G4-Mid, and G5-High doses) compared directly against the mean baseline values of their respective vehicle-treated disease control groups (G2-Control). Spatial protein localization and accumulation were quantified via IHC H-scores. Transcriptional biomarker expression levels were assessed via qRT-PCR and originally measured as C_t_ values. Because an increase in C_t_ value inversely correlates with target mRNA expression, the relative change for qRT-PCR data was inverted to reflect true biological shifts. Consequently, for PCR data, yellow hues denote a statistically significant biological upregulation or accumulation of the target biomarker, whereas green hues indicate a protective downregulation relative to the disease baseline. LFCM, lung fibroblast conditioned medium; IHC, immunohistochemistry; qRT-PCR, quantitative reverse transcription polymerase chain reaction; Ct, cycle threshold.

Conversely, in models characterized by dense collagenous scarring, LFCM intervention appeared to actively promote ECM clearance pathways. In the bleomycin-induced fibrosis model, this was characterized by a statistically significant elevation in MMP-12 H-scores within the G4-Mid and G5-High dose groups, coupled with notable increases in Cathepsin-D expression (G3-Low and G5-High doses, Figure 5, Table S8).

The particulate models exhibited highly specific responses: the silica model demonstrated a noticeable decrease in Cathepsin-D (G5-High) paired with notable increases in MMP-12 across all doses. Strikingly, aligning with the anomalous histopathological and gross tissue observations previously noted, the asbestos G5-High dose group demonstrated a statistically significant increase in both Cathepsin-D and α-SMA H-scores, further substantiating a localized hypersensitivity or pro-fibrotic activation at the highest dose in this specific lung injury pathway (Figure 5).

#### 3.5.2. Transcriptional Suppression of Pro-Fibrotic Mediators

Quantitative reverse transcription PCR (qRT-PCR) was employed to evaluate the pre-translational suppression of key fibrogenic markers. In this analysis, an increase in the cycle threshold (C_t_) value inversely correlates with target mRNA expression, denoting the active transcriptional downregulation of the target gene.

LFCM treatment drove a robust, broad-spectrum transcriptional suppression of fibrillar collagen and myofibroblast activation markers. Significant pre-translational downregulation of the gene encoding collagen type III (evidenced by significant increases in C_t_ values) was consistently observed across the hyperoxia (G4-Mid and G5-High), LPS (G5-High), asbestos (G4-Mid and G5-High), and bleomycin (G5-High) models relative to their respective positive controls.

Furthermore, LFCM significantly inhibited the upstream transcriptional drivers of tissue remodeling. Significant transcriptional suppression of the gene encoding TGF-β1 was recorded in the LPS and silica models (all specifically within the G4-Mid and G5-High dose cohorts). Concurrently, significant pre-translational downregulation of the gene encoding α-SMA, the primary molecular signature of myofibroblast transdifferentiation, was successfully achieved in the hyperoxia (G4-Mid), LPS (G4-Mid and G5-High), and bleomycin (G3-Low, G4-Mid, and G5-High) dose groups, confirming that LFCM actively halts fibrotic progression at the genomic level. Finally, while upregulation of the gene encoding collagen I was achieved by the G3-Low dose in the hyperoxia and asbestos arms, downregulation was noted for G5-High in the silica model and for G4-Mid and G5-High in the bleomycin model (Figure 5).

## 4. Discussion

### 4.1. Broad-Spectrum Efficacy of FE002-Lu LFCM and the Developmental Paradigm of Tissue Repair

The present study systematically evaluated the therapeutic efficacy of intratracheally administered FE002-Lu LFCM across five mechanistically distinct in vivo rodent models of acute and chronic pulmonary injury: hyperoxia, LPS, asbestos, silica, and bleomycin [24]. A central novelty of this intervention lies in its biological ontogeny. Specifically, the therapeutic secretome was derived from the primary FE002-Lu cell type, a highly standardized and cultured fetal lung fibroblast population originally sourced from a single, rigorously documented 14-week-gestation organ donation in Switzerland [15]. At 14 weeks of gestation, the human fetal lung is in the highly proliferative pseudoglandular stage of development, an ontogenetic window characterized by active branching morphogenesis, profound intrinsic immunotolerance, and a unique, transient capacity for scarless tissue repair [15,46,47].

The experimental data demonstrate that FE002-Lu LFCM exerts a potent, dose-dependent protective effect across this diverse mechanistic spectrum. Specifically, macroscopic preservation and significant reductions in Ashcroft scores were achieved across the hyperoxia, LPS, silica, and bleomycin models following the 28-day dosing regimen. This broad-spectrum efficacy suggests that the complex network of paracrine factors contained within this specific fetal secretome retains critical developmental cues. Thus, rather than acting through a single molecular target, FE002-Lu LFCM acts pleiotropically to override pathological adult wound-healing mechanisms, effectively working toward transitioning the pulmonary microenvironment from a state of progressive, irreversible structural degradation toward a resolving, functional state [7].

Of note, the reported robust efficacy of the investigated FE002-Lu secretome further underscores the distinct biological advantages of fetal-derived progenitor cells over conventional adult MSC therapies [8,9,16]. While adult MSCs are inherently susceptible to cumulative epigenetic drift, profound donor-to-donor variability, and the rapid onset of a senescence-associated secretory phenotype (SASP) during in vitro expansion, primary fetal fibroblasts maintain superior genomic stability and robust telomerase activity [15,48,49]. Consequently, EVs and soluble fractions composing the LFCM are most certainly enriched with early-developmental morphogens, pleiotropic growth factors, and pro-regenerative microRNAs [8,46]. This unique cargo is natively programmed to orchestrate scarless organogenesis and tissue tolerance, providing a distinct functional advantage over adult-derived secretomes that are intrinsically primed for mature, fibrotic scar formation [9,47].

Beyond the ontogenetic advantages of fetal cells, the organ-specific origin of the FE002-Lu secretome likely provides a ‘homing’ of biochemical cues [16]. Since these cells are natively programmed for pulmonary homeostasis, their secretome may contain a specific ratio of matrix-modulating enzymes (MMPs/TIMPs) that generic mesenchymal sources lack, tailoring the local and homologous intervention to the unique architectural requirements of the lung. Of note, although the specific molecular composition of the FE002-Lu secretome requires further elucidation, human fetal lung fibroblast secretomes are natively rich in highly potent immunomodulatory and anti-fibrotic mediators [9,50]. Factors such as hepatocyte growth factor (HGF) and prostaglandin E_2_ (PGE_2_), for instance, are well-documented drivers of M2 macrophage polarization and potent negative regulators of the TGF-β1 signaling cascade [51]. While the broad-spectrum efficacy demonstrated herein strongly suggests that these, or similarly pleiotropic early-developmental morphogens, are the active agents mediating the profound phenotypic shifts across the evaluated LI models, our current data reflect observed downstream biological responses rather than direct molecular receptor antagonism. Thus, ongoing and future proteomic and transcriptomic deconvolution of the FE002-Lu secretome are required to definitively identify these active agents and map their precise binding interactions.

Compared to current FDA-approved anti-fibrotics such as nintedanib and pirfenidone, which primarily target isolated tyrosine kinase receptors or TGF-β synthesis to decelerate decline, the FE002-Lu LFCM offers a distinctively pleiotropic advantage [1,6,8,52,53,54]. In our bleomycin-induced model, FE002-Lu LFCM not only reduced TGF-β1 and collagen deposition but also concurrently modulated MMP-12-mediated matrix turnover and stabilized lysosomal activity. This multifaceted efficacy, broadly targeting inflammation, myofibroblast activation, and active ECM clearance simultaneously, contrasts with the single-target pharmacotherapies that frequently fail to reverse established architectural distortion or significantly reduce mortality in end-stage ILDs [1,6,8,53,54].

Furthermore, beyond superior pleiotropic efficacy, the localized, cell-free nature of the FE002-Lu LFCM addresses a major hurdle in clinical pulmonology: patient compliance and quality of life (QoL). In detail, current oral anti-fibrotics are frequently associated with severe systemic toxicities, including profound gastrointestinal distress, photosensitivity, and hepatotoxicity, leading to high rates of treatment discontinuation [1,52,54]. By locally delivering the therapeutic secretome directly to the compromised alveolar epithelium via inhalation, FE002-Lu LFCM has the potential to maximize target-site bioavailability while significantly minimizing systemic exposure [55]. This targeted approach theoretically circumvents the debilitating off-target side effects characteristic of systemic oral therapies, offering a far more tolerable regimen for patients requiring life-long management of progressive fibrotic diseases.

Notwithstanding, despite these compelling in vivo outcomes, the FE002-Lu LFCM currently remains a complex biological composite. Specifically, to transition from a phenomenological observation study to a precisely characterized biologic, it is imperative to explicitly deconvolute this cell-derived ‘black box’ [56]. However, the already wide scope of this specific study was the in vivo phenotypic and functional validation of the unfractionated FE002-Lu secretome, which now serves as the foundational justification for future, isolated EV-fraction analyses. Thus, building upon this validated physiological baseline, our subsequent analytical milestones will focus on explicitly deconvoluting this cell-derived secretome through deep proteomic mapping via liquid chromatography–mass spectrometry (LC-MS) and transcriptomic sequencing to isolate and characterize the primary active components. Future efforts must therefore scrutinize the EV cargo to identify the exact concentration of pro-resolving microRNAs and soluble cytokines [8,55]. Specifically, by isolating the EV fractions from the soluble protein fractions and testing them independently in robust primary human lung platforms, we further aim to isolate the primary active pharmaceutical ingredients driving this broad-spectrum efficacy [3,57].

### 4.2. Paracrine Immunomodulation and the Push Toward Resolution of the Cytokine Storm

A fundamental hallmark of acute pulmonary injury, regardless of the initiating etiology, is the rapid induction of a localized cytokine storm. The FE002-Lu secretome demonstrated a strong capacity to truncate this hyperinflammatory cascade. In the LPS infectious model and the hyperoxia oxidative model, significant reductions in IL-1β, IL-6, TNF-α, and the neutrophil chemoattractant CINC-1 in the BALF of mid- or high-dose groups indicated a robust suppression of the innate acute-phase response [5,12,30,31,32,34,50,58]. This suppression mirrors existing in vivo data, where human umbilical cord-derived MSCs successfully attenuated localized and systemic inflammation within a rat model of endotoxin-induced injury [21]. However, the authors only observed effects over 48 h and strikingly observed no effect in the MRC-5 control group [21]. Crucially, the observed biomarker suppression in our datasets was consistently coupled with a significant, dose-dependent elevation in the regulatory cytokine IL-10. This distinct molecular shift indicates that the studied fetal secretome actively reprograms the local immune milieu toward an immunosuppressive, pro-resolution phenotype [9,45,49].

Mechanistically, this robust elevation in IL-10 strongly suggests that the FE002-Lu LFCM drives a phenotypic switch in the resident alveolar macrophage population [59]. By truncating the initial innate response, the secretome likely promotes the transition of macrophages from a classical, tissue-damaging M1-like state toward an alternatively activated, pro-resolution M2-like phenotype [4,10,12,25,33,35,59]. This repolarization is critical, as M2 macrophages are primary orchestrators of debris clearance and the dampening of destructive polymorphonuclear neutrophil extravasation [5,29,60]. The robust, dose-dependent elevation in IL-10 across disparate models, from infectious LPS to oxidative hyperoxia, suggests that FE002-Lu LFCM acts as a molecular switch for the innate immune system [37]. In detail, by promoting an M2-like phenotype, FE002-Lu LFCM likely enhances the clearance of cellular debris and prevents the chronic activation cycles that typically characterize frustrated phagocytosis in the presence of biopersistent particulates.

Furthermore, our data indicates that localized FE002-Lu LFCM administration regulates the systemic immune response [61]. For example, the asbestos model triggered the broadest systemic hematological activation, evidenced by elevated peripheral WBC, lymphocyte, and eosinophil counts. Of note, FE002-Lu LFCM effectively modulated this crosstalk, particularly stabilizing the alveolar–capillary barrier [3,12]. Indeed, the significant reduction in BALF platelet counts in this cohort suggests a restoration of endothelial integrity, thereby reducing vascular leakage and limiting the pathological extravasation of circulating thrombotic effectors into the lung parenchyma [3,5,10,61,62,63]. While this restored barrier restricts the infiltration of circulating effectors, the concurrent systemic upregulation of innate immune cells suggests a complex systemic-local crosstalk [5,30]. It remains to be elucidated whether the intratracheally applied FE002-Lu LFCM diffuses across the stabilized endothelial barrier into systemic circulation to mobilize bone marrow reserves directly, or if it acts locally to stimulate resident alveolar cells to secrete secondary systemic chemokines, though recent pharmacokinetic data heavily favor the latter [4,61,64].

The observed reduction in IL-1β levels across the particulate and oxidative models is particularly significant, as it suggests that FE002-Lu LFCM may actively inhibit the NLRP3 inflammasome [25]. In clinical scenarios of asbestosis and silicosis, frustrated phagocytosis by alveolar macrophages triggers a persistent inflammasome-mediated release of IL-1β, driving a self-perpetuating loop of chronic inflammation [35,65,66]. By suppressing this pathway while simultaneously elevating IL-10, FE002-Lu LFCM appears to facilitate a ‘molecular reset’ of the macrophage phenotype [66]. As stated before, this likely transitions the microenvironment from a pro-inflammatory M1-like state toward an alternatively activated M2-like phenotype natively programmed for debris clearance and tissue homeostasis, thereby addressing the root cause of particulate-induced injury rather than merely masking the symptoms [59,60,63].

Importantly, the administration of a human-derived biologic to an immunocompetent rodent model inherently introduces a xenogeneic barrier, which warrants careful contextualization. As observed in our quantitative cellular profiling, LFCM treatment did upregulate specific localized and systemic innate immune cell counts (e.g., increased BALF monocytes in the hyperoxia arm and elevated systemic neutrophils/monocytes in the LPS arm). However, a classical xenogeneic immune rejection, or an adverse inflammatory reaction to the human vesicles, would characteristically manifest as an uncontrolled, exacerbated cytokine storm. Instead, our biochemical data demonstrate that this cellular mobilization occurred precisely concurrently with a strong, statistically significant suppression of key pro-inflammatory drivers (IL-1β, IL-6, TNF-α, and CINC-1) and a marked elevation in the potent anti-inflammatory cytokine IL-10. Therefore, we postulate that the observed cellular increases do not reflect a pathological immune rejection of the human secretome. Rather, the simultaneous rise in specific innate immune cells and IL-10 strongly aligns with a secretome-guided immunomodulation, specifically the targeted recruitment and phenotypic switching of macrophages toward an alternatively activated, pro-resolution M2-like state. This guided cellular influx is functionally essential for orchestrating targeted debris clearance and transitioning the pulmonary microenvironment toward structural resolution, a hypothesis corroborated by the ultimate histological preservation and lack of severe procedural toxicity observed across the treated cohorts.

### 4.3. Downstream Modulation of the TGF-β1/WNT Axis and Myofibroblast Deactivation

The capacity for scarless healing inherent to early fetal tissue was most evident in FE002-Lu LFCM’s ability to arrest fibrotic progression. Generally, TGF-β1 serves as the canonical master regulator of pulmonary fibrosis, driving the transdifferentiation of quiescent resident fibroblasts into matrix-secreting myofibroblasts [6,25,37,65,67]. Treatment with FE002-Lu LFCM resulted in downstream suppression of this axis, a phenomenon increasingly documented in therapeutic EV applications, evidenced by significantly reduced overall TGF-β1 tissue levels alongside corresponding decreases in tissue inhibitor of metalloproteinases-1 (TIMP-1) and HYP in tissue homogenates [7,10,55,68]. While the specific molecular-level interactions driving this suppression remain to be deconvoluted, at the cellular level, this functional anti-fibrotic effect was confirmed by significant decreases in the protein expression of α-SMA [66,69].

Furthermore, the FE002-Lu secretome demonstrated vital epithelial-protective effects by significantly downregulating WNT1-inducible signaling pathway protein-1 (WISP-1) across models, a critical matricellular mediator that promotes epithelial-to-mesenchymal transition (EMT) following alveolar stress [6,43,70,71]. Specifically, WISP-1 functions as a crucial downstream effector of the highly conserved WNT/β-catenin signaling cascade [70,71]. By downregulating this pathway, FE002-Lu LFCM likely protects the critical population of alveolar epithelial type II (AECII) progenitor cells from undergoing pathological mesenchymal transdifferentiation [41,42,70]. Importantly, this capacity is a hallmark of stem cell-derived vesicles; in vitro dosing from human adipose stem cells has been shown to comprehensively rescue cellular proliferation rates even when cells are under direct LPS-induced inflammatory stress or active peroxide-driven oxidative damage [14]. Critically, preserving the functional AECII pool is imperative not only for maintaining the physical integrity of the alveolar–capillary barrier but also for sustaining pulmonary surfactant production, a function that is catastrophically lost during the architectural collapse and terminal honeycombing characteristic of end-stage fibrotic disease [26]. These structural observations were robustly reinforced at the pre-translational level, where qRT-PCR data confirmed that FE002-Lu LFCM strongly suppresses the mRNA transcription of collagen type III and α-SMA, effectively halting the fibrotic cascade at the genomic level prior to mature protein synthesis [6,72].

### 4.4. Biphasic Regulation of Macrophage Proteostasis and ECM Turnover

A sophisticated, context-dependent feature of this fetal cell-derived therapy is its biphasic modulation of proteolytic enzymes, specifically macrophage metalloelastase (MMP-12) and Cathepsin-D [62,63]. In acute, highly destructive inflammatory environments (such as the LPS and hyperoxia models), recruited macrophages most probably adopt an aggressive phenotype, secreting MMPs that cause collateral degradation of the functional lung matrix [2,7,26,27,38,73]. In these cohorts, FE002-Lu LFCM reduced Cathepsin-D and MMP-12 expression (although not statistically significant), suggesting that the secretome effectively stabilizes lysosomal membranes and dampens excessive macrophage proteolysis to preserve the native ECM scaffold [2,10,41,74,75,76].

Conversely, in the bleomycin model, which captures the active fibroproliferative phase of early collagen deposition, FE002-Lu LFCM treatment concurrently downregulated pro-fibrotic markers while significantly increasing the expression of MMP-12 [43]. This highly dynamic dual response implies that the fetal secretome does not simply arrest new scar formation; it actively facilitates the targeted enzymatic turnover and mitigation of newly deposited pathological ECM during this critical early-remodeling window [7,67,73,77,78]. Moderately elevated Cathepsin-D in this context points toward enhanced lysosomal autophagy, suggesting that FE002-Lu LFCM reprograms local macrophages into an alternative pro-resolution phenotype capable of early matrix modulation [47,59,60,76,77,79,80].

### 4.5. LFCM Dose Optimization and Model-Specific Sensitivities

The comprehensive experimental data presented herein systematically establish a distinct therapeutic window for FE002-Lu LFCM across disease etiologies, which strengthens their significance [44]. While treatment demonstrated clear, dose-dependent therapeutic benefits, the mid-dose frequently provided the most consistent, balanced, and comprehensive protection across all measured inflammatory and fibrotic parameters, which is not uncommon for biologics and requires iterative fine-tuning [68,81,82]. Notably, in complex particulate-induced models (specifically asbestos), the highest dose triggered a paradoxical increase in Ashcroft scores, total collagen deposition, and localized Cathepsin-D and α-SMA expression. This anomaly suggests a potential localized hypersensitivity or secretome saturation effect when high concentrations of bioactive trophic factors interact directly with persistently retained physical asbestos fibers. In such extreme microenvironments, the overwhelming influx of secretome factors may overstimulate local myofibroblasts and dysregulate ECM turnover, inadvertently reinforcing fibrotic signaling rather than resolving it [83]. Indeed, this potential for the secretome to paradoxically exacerbate disease through aberrant ECM remodeling and fibroblastic switching has similarly been recognized as a critical risk factor in other severe pathologies, such as tumor progression [84].

In detail, unlike pharmacological or oxidative triggers, asbestos fibers possess a unique biopersistence that prevents enzymatic degradation, locking resident macrophages in a perpetual state of frustrated phagocytosis and chronic NLRP3 inflammasome activation [35,36]. This prolonged, incomplete clearance saturates the local microenvironment with ROS and pro-inflammatory danger signals like HMGB1, fueling a relentless and highly reactive inflammatory state [85]. We hypothesize that introducing fetal-derived proliferative factors (intended for rapid organogenesis) into a microenvironment dominated by inescapable physical irritation and unchecked ROS generation may inadvertently fuel aberrant fibroproliferation [2,26,28,86]. Specifically, an overabundance of trophic factors at the highest dose might hyper-stimulate macrophage phagocytic activity. In a typical microenvironment, this enhanced activity facilitates debris clearance; however, against biopersistent asbestos fibers, this hyper-stimulation likely leads to catastrophic lysosomal membrane rupture [36,87]. This rupture would spill elevated Cathepsin-D and α-SMA, which were significantly increased in the high-dose asbestos cohort, into the cytosol, thereby exacerbating NLRP3 inflammasome activation and driving the paradoxical fibroproliferative response observed [76,88,89]. In this distinct context, an overabundance of growth factors likely synergizes with the unresolved inflammatory loops (fueling a ‘runaway’ proliferative response), exacerbating rather than resolving the fibrotic cascade [81].

Consequently, while the high dose bears the potential to be curative in chemical or oxidative models, a mid-dose tier likely represents the optimal safety–efficacy balance for treating injuries involving permanent physical particulates. Furthermore, the mid-dose emerges as the optimal, scientifically validated candidate for advancing toward human clinical applications, offering maximum anti-fibrotic efficacy while avoiding hyper-reactive particulate responses.

### 4.6. Advanced Formulation and the Rationale for Inhalation-Based Therapy

From a translational and pharmacological perspective, the specific formulation of the investigated biologic is a critical attribute. Traditional live-cell therapies (such as adult MSC engraftment) are chronically hindered by stringent cryopreservation requirements, complex cold-chain logistics, and the physical fragility of live cells [84]. In this study, the FE002-Lu LFCM was advanced into a lyophilized (freeze-dried) powder housed within sterile vials, rendering it a highly stable, “off-the-shelf” biologic capable of worldwide distribution under uncontrolled environmental conditions [11,48,53,56,90]. This carries profound implications for global health equity. By eliminating the requirement for liquid nitrogen storage or complex cold-chain logistics, this formulation is well-suited for rapid deployment in low-resource settings where occupational hazards, such as unregulated silica or asbestos exposure, are often most prevalent [91]. The functional stability of the secretome’s components ensures that the therapeutic potency is preserved across varied environmental conditions, establishing a viable pathway for high-impact outpatient care (such as targeted nebulization) and industrial accident response in diverse geographic regions [92,93]. Crucially, transitioning to a freeze-dried layout may offer distinct biological advantages over traditional liquid cryostorage. Extended preservation at −80 °C has been shown to induce severe, cumulative structural degradation, resulting in physical vesicle aggregation and a noticeable loss of vital surface proteins over time, deleterious alterations that are entirely neutralized by stable lyophilization matrices [14]. Furthermore, the robust, broad-spectrum in vivo efficacy demonstrated throughout this study, spanning the suppression of acute cytokine storms to working toward the halting of fibrotic progression, serves as functional proof that this specific lyophilization matrix successfully preserves the structural integrity and biological potency of the secretome’s extracellular vesicles and soluble mediators upon reconstitution.

Crucially, because FE002-Lu LFCM is entirely cell-free, it is uniquely suited for non-invasive respiratory administration [94,95]. While i.t. instillation was necessary for controlled dosing in the present rodent models, this route is invasive and impractical for routine clinical care [64]. The robust nature of the lyophilized secretome means it can be easily reconstituted in a sterile vehicle at the point of care and administered via clinical nebulizers (e.g., vibrating mesh or jet nebulizers) [13]. Unlike live cells, which are instantly sheared and destroyed by the mechanical forces of aerosolization, the nanoscale components of the FE002-Lu LFCM are highly likely to withstand nebulization [10,95]. Importantly, a nebulized formulation would allow for uniform, bilateral distribution of the therapeutic substance directly to the deep alveolar epithelium, dramatically improving patient compliance, enabling at-home outpatient administration, and optimizing the localized bioavailability in humans [12,14,62,96,97]. This capacity of lyophilized, room-temperature-stored vesicles to retain functional potency upon point-of-care reconstitution is well-supported by recent work in neuronal injury models, where rehydrated sEVs successfully enhanced cell migration rates and stimulated wound healing [14].

Notwithstanding, translating this therapy from the current experimental delivery method to clinical nebulization will require bridging a significant pharmacokinetic gap. Specifically, the i.t. instillation utilized in this study delivers the secretome as a localized liquid bolus, which is subject to gravitational pooling and distinct regional clearance rates [98]. In contrast, clinical nebulization delivers a continuous aerosol with a highly uniform aerodynamic mass median diameter (AMMD), resulting in fundamentally different alveolar deposition fractions and local tissue half-lives [95,99]. Thus, subsequent large-animal preclinical trials shall utilize standardized vibrating mesh nebulizers to accurately map the aerosolized biodistribution, confirm deep lung penetration, and establish precise pharmacokinetic dosing equivalents prior to human translation [64,100,101].

For clinical implementation, the nebulized delivery of FE002-Lu LFCM offers highly versatile integration into existing medical workflows. In the intensive care unit (ICU), FE002-Lu LFCM could be seamlessly administered via in-line vibrating mesh nebulizers directly into the ventilator circuits of patients suffering from ARDS, ensuring deep alveolar penetration even during positive pressure mechanical ventilation [4,102,103]. Conversely, for the management of chronic fibrotic conditions such as IPF, the lyophilized biologic can be safely reconstituted by the patient at home and administered via standard handheld ultrasonic nebulizers. This dual-track delivery potential maximizes FE002-Lu LFCM’s clinical utility across both emergency critical care and long-term outpatient pulmonology settings [104].

From a pharmacoeconomic perspective, this dual-track delivery system presents a compelling value proposition for healthcare payers and health systems. In the acute setting, the rapid mitigation of the cytokine storm and stabilization of the alveolar–capillary barrier could significantly reduce the duration of mechanical ventilation and total ICU bed days, two of the most substantial cost drivers in critical care medicine [4,33,105,106]. Recent clinical data validating nebulized mesenchymal stem cell secretomes (from adult human adipose stem cells) in severe ARDS cohorts have already demonstrated measurable decreases in total ICU stay durations, confirming the economic feasibility of this approach [106,107]. Furthermore, the ability to seamlessly transition chronic patients to at-home, self-administered nebulized therapy minimizes the need for recurrent outpatient hospital visits and invasive intravenous infusions [92]. By fundamentally modifying disease progression rather than merely managing symptoms, FE002-Lu LFCM holds the potential to delay or entirely avert the need for high-cost end-stage interventions, such as lung transplantation, thereby alleviating a massive economic burden on global healthcare infrastructures [1,8,54].

Furthermore, the pleiotropic nature of FE002-Lu LFCM suggests its potential as a powerful adjunct to current clinical standards of care. In the context of infectious triggers like LPS or severe pneumonia, FE002-Lu LFCM could be administered alongside systemic antibiotics to dampen the destructive cytokine storm that often persists even after bacterial clearance, while simultaneously enhancing bacterial clearance mechanisms [5,57,102]. Similarly, in ARDS patients requiring life-saving but potentially toxic 100% oxygen therapy, the antioxidant and epithelial-protective effects demonstrated in our hyperoxia model could mitigate the secondary oxidative damage associated with prolonged ventilation [2,26,27,28]. This capability to serve as a disease-modifying bridge during acute crises significantly broadens the therapeutic scope for FE002-Lu LFCM in critical care settings.

### 4.7. Study Limitations

While the multi-model design of this study provides robust evidence of FE002-Lu LFCM’s broad-spectrum efficacy, certain limitations must be acknowledged. First, while these rodent models accurately mimic the acute inflammatory and early fibrotic phases of human lung disease, they do not perfectly replicate the protracted chronicity of human interstitial lung diseases like IPF, which typically progress over several years [3,8,12,24,44,62,107]. Furthermore, while 100% survival was maintained across all experimental arms, this likely reflects a specific baseline severity characteristic of mild-to-moderate early-stage clinical disease [24]. Given that clinical ARDS and progressive IPF carry substantial mortality burdens, the boundaries of FE002-Lu LFCM’s rescue capabilities in terminal, end-stage respiratory failure remain to be fully defined [1,12,69]. Additionally, the 28-day evaluation window, while standard for pre-clinical toxicology and efficacy screening, limits the assessment of long-term tissue remodeling, late-onset toxicity, or potential relapse following the cessation of therapy [22,25,108].

To bridge this translational gap and fully validate the effect durability of FE002-Lu LFCM, future pre-clinical study designs must incorporate extended longitudinal tracking. Extending the observation period to 3–6 months post-injury will be critical to rigorously evaluating the permanence of the observed beneficial effects on fibrotic drivers [108,109]. Furthermore, these longitudinal cohorts will allow for the assessment of potential delayed-onset localized toxicities, secondary immune sensitizations, and whether the targeted pulmonary microenvironment maintains its pro-resolution trajectory or demonstrates fibrotic relapse upon complete withdrawal of the therapeutic secretome [10,110]. The anomalous pro-fibrotic response observed uniquely in the high-dose asbestos group also warrants dedicated, long-term toxicological unpacking to ensure patient safety in targeted occupational exposure cohorts.

A second notable limitation of the current experimental design is the exclusive utilization of male Wistar rats [44]. While this narrows global host endocrine translatability, choosing a highly standardized male Wistar background provides a controlled, universally recognized baseline for tracking acute pulmonary oxygen toxicity for example, matching historical dose–response and oxidant/antioxidant imbalance data in the literature [20,26]. However, it is well-documented in pulmonary research that sex hormones, particularly estrogen, exert baseline protective effects against fibrogenesis and oxidative LI, potentially altering the baseline trajectory of the inflammatory response [111,112]. Estrogen is known to independently suppress TGF-β1 signaling, attenuate pro-inflammatory macrophage activation, and limit pathological ECM deposition. Consequently, by omitting female subjects, the present study assesses FE002-Lu LFCM exclusively in a microenvironment where fibrotic susceptibility is inherently maximized due to the absence of endogenous estrogenic protection. This specific design impacts our conclusions regarding universal dose optimization: it remains unknown whether the optimal mid-tier dose established here would be redundant, synergistic, or excessively immunosuppressive when interacting with the natively protective endocrine profile of a female host. Furthermore, because IPF and occupational lung diseases exhibit distinct sex-based disparities in human clinical prevalence and progression, with over 70% of IPF diagnoses occurring in males who subsequently demonstrate poorer survival trajectories, future preclinical investigations must incorporate female cohorts [1,111,112]. Evaluating FE002-Lu LFCM across both sexes will be imperative to confirm that its pleiotropic efficacy and dose–response dynamics are not heavily influenced by host endocrine profiles.

A third specific methodological caveat lies in the utilization of the single-dose intratracheal bleomycin LI model. While it remains the gold standard for evaluating early fibrogenic pathways, rodent bleomycin-induced fibrosis inherently tends toward spontaneous self-resolution beyond the 28-to-35-day post-instillation mark, fundamentally diverging from the relentless, self-perpetuating chronicity of human IPF [24,25,43,45]. Therefore, while our data show that FE002-Lu LFCM works toward effectively halting the active fibroproliferative phase, subsequent studies must employ repetitive bleomycin dosing regimens or aging transgenic murine models (e.g., telomerase-deficient mice) that genuinely mimic the progressive, non-resolving pathological environment of end-stage human interstitial lung disease [6,45,113].

Furthermore, while this study utilized robust histological scoring (e.g., Ashcroft scores) and frequent clinical observation to approximate respiratory preservation, direct in vivo pulmonary function tests (e.g., plethysmography or invasive lung mechanics) were not performed. Future advanced preclinical evaluations must therefore incorporate precise physiological respiratory measurements to definitively correlate the observed molecular and structural improvements with functional aerodynamic recovery. Additionally, because this study prioritized broad-spectrum structural and inflammatory screening across five diverse etiologies, exhaustive pathway mapping for individual models was not performed. For instance, while we demonstrated significant reductions in localized NO within the hyperoxia model, future dedicated studies must incorporate a comprehensive panel of classical oxidative stress markers (e.g., MDA, GSH, SOD, and catalase) to fully elucidate the specific antioxidant mechanisms driven by the FE002-Lu secretome. Furthermore, because the analytical framework of this broad-spectrum study prioritized downstream cytokine networks and macro-level structural remodeling, the direct intracellular signaling mechanisms remain to be fully elucidated. Future dedicated molecular studies must specifically investigate canonical intracellular pathways, such as NF-κB translocation for acute inflammation, Smad2/3 phosphorylation for TGF-β1-driven fibrogenesis, and the Nrf2 pathway for oxidative stress, to definitively validate the precise cellular mechanisms of action exerted by the FE002-Lu secretome.

Furthermore, the timing of therapeutic intervention in this study must be carefully contextualized. Our protocol initiated FE002-Lu LFCM treatment shortly after the onset of clinical symptoms, a window highly relevant for acute presentations like ARDS, chemical spills, or early-stage exacerbations where active inflammation and myofibroblast transdifferentiation are still dynamic [45]. However, clinical interstitial lung diseases such as IPF are frequently diagnosed at an advanced stage, characterized by dense, established, and largely acellular collagen matrices. While FE002-Lu LFCM demonstrated an encouraging capacity to upregulate matrix-clearing enzymes like MMP-12 in established bleomycin scars, establishing the absolute efficacy of FE002-Lu LFCM in reversing highly mature, long-standing human fibrotic lesions will require further evaluation in delayed-intervention, late-stage animal models [39,45].

A final methodological limitation of the present study design is the composition of the vehicle control. While the therapeutic FE002-Lu LFCM was formulated with equimolar concentrations of sucrose and trehalose to ensure biological stability during lyophilization, the control cohorts received standard physiological saline [13]. Although both of these disaccharides are generally recognized as safe (GRAS) and are standard inert excipients in inhalation therapies, they can exert mild, transient localized osmotic effects in the respiratory tract [114]. Importantly, the inclusion of these specific sugars is structurally vital, as freeze-drying cell-derived secretomes in un-stabilized saline or phosphate buffers has been shown to result in extensive vesicle destruction and membrane shearing [13]. While the robust, dose-dependent anti-fibrotic and immunomodulatory effects observed heavily implicate the active secretome cargo as the primary therapeutic driver, future Investigational New Drug (IND)-enabling studies must utilize an exact excipient-matched vehicle control. This rigorous control will be essential to definitively isolate the biological efficacy of the FE002-Lu secretome from any minor baseline physicochemical effects imparted by the cryoprotectant matrix.

### 4.8. Future Perspectives and Clinical Translation

The demonstrated efficacy of FE002-Lu LFCM against biopersistent particulates also opens a novel avenue for occupational medicine: secondary prophylaxis. Currently, following acute industrial exposure to high concentrations of silica or asbestos, clinical intervention is largely observational until irreversible radiographic or symptomatic changes manifest [65]. Because FE002-Lu LFCM acts rapidly to reprogram macrophages and halt early frustrated phagocytosis before dense collagenous networks form, it could be stockpiled by industrial safety agencies as a rapid-response medical countermeasure [36,65]. Thus, future studies could investigate the prophylactic administration of low-to-mid-dose FE002-Lu LFCM immediately following documented acute occupational exposure events to determine if early intervention can fully abort the chronic fibrotic cascade before clinical silicosis or asbestosis develops.

Although the 28-day repeated dosing regimen yielded 100% survival without immediate procedural toxicity, both clinical regulators and prescribing physicians will require a comprehensive profiling of long-term immunogenicity [114,115]. Because FE002-Lu LFCM is an allogeneic, human-derived biologic, the potential for the host immune system to generate anti-drug antibodies (ADAs) following chronic respiratory administration remains a critical translational question [115]. Future longitudinal studies must rigorously evaluate systemic and localized ADA formation to confirm that repeated nebulization does not induce neutralizing immune responses or secondary hypersensitivity, thereby safeguarding long-term patient compliance and therapeutic durability [11,114,115].

Importantly, while FE002-Lu LFCM is administered locally to the respiratory tract, our hematological data confirm that it exerts modulatory effects on the systemic immune profile. This systemic crossover highlights the necessity for comprehensive, Good Laboratory Practice (GLP)-compliant toxicological profiling [92]. Given that the FE002-Lu LFCM is most probably enriched with potent, early-developmental proliferative factors, future safety evaluations must include rigorous histopathological assessments of distal filtering organs (specifically the liver, kidneys, and spleen) where systemic clearance and accumulation occur [64,92]. Confirming the absence of off-target hyperplastic or pro-oncogenic effects in these distal tissues will be a mandatory regulatory milestone to validate the long-term systemic safety profile of this investigational inhaled biologic [11,116].

Furthermore, translating the efficacy of a human-derived biologic using an immunocompetent rodent model inherently introduces a xenogeneic barrier [117]. However, the successful deployment of human perinatal-derived cellular therapies in fully immunocompetent rat models of acute LI is well-precedented in respiratory literature, demonstrating that conserved protective mechanisms can effectively operate across this cross-species barrier [21]. While the highly conserved nature of critical early-developmental morphogens likely facilitated the robust cross-species efficacy observed in the reported 28-day window, the repeated administration of human proteins into a fully immunocompetent rat host inevitably triggers a neutralizing immune response over time. Thus, it is plausible that the therapeutic potency of FE002-Lu LFCM was partially blunted by cross-species immune clearance in the later stages of the protocol. Consequently, the true ceiling of FE002-Lu LFCM’s efficacy may be underestimated in this Wistar rat model, highlighting the need for future homologous (rat-to-rat) or humanized-mouse models to accurately map the absolute pharmacokinetic durability of the secretome without xenogeneic confounding [118,119].

Nevertheless, the future clinical perspectives for this investigational biologic are highly promising. From a safety and regulatory perspective, the primary FE002-Lu primary cell type shares profound biological and manufacturing similarities with the historically and compendially validated MRC-5 and WI-38 human diploid fibroblast cell types [16]. In detail, these legacy primary fetal cell types have been safely and extensively utilized worldwide for decades as highly stable substrates for human vaccine production, with a thoroughly documented absence of neoplastic or tumorigenic safety issues. Aligning directly with this established paradigm dating back to the Hayflick era, the FE002-Lu cells may continue to be industrially cultivated across multiple jurisdictions (e.g., Switzerland, Singapore, China) under a strictly regulated, GMP-compliant framework and exhibit an inherently high biosafety profile [15,16].

A critical corollary of this methodology is its immense industrial sustainability. By relying on a single, stringently characterized and qualified 14-week-gestation organ donation, this approach entirely circumvents the variability of pooled adult donor cells. Despite possessing a finite in vitro lifespan governed by the Hayflick limit, the highly controlled expansion of this single diploid cell source into tiered Master and Working Cell Banks yields a level of batch-to-batch consistency and sustainability that is practically infinite [15]. This single-donor biobanking strategy thus ensures a highly sustainable, standardized therapeutic yield capable of fueling decades of global commercial production, effectively streamlining the regulatory pathway for FE002-Lu LFCM compared to de novo biologics [15].

Nevertheless, while the single-donor FE002-Lu biobanking strategy ensures foundational batch-to-batch consistency, successful industrial translation will potentially require transitioning this robust biological platform from 2D adherent cultures to highly scalable 3D bioreactor systems [10,11,65,68]. Therein, defining the precise upstream hydrodynamic shear stress and dissolved oxygen parameters that optimize the cellular secretion of anti-fibrotic EVs versus soluble proteins will be critical [11,120]. Establishing these scalable upstream parameters, coupled with high-yield downstream processing techniques like tangential flow filtration (TFF), will be necessary to achieve the commercial viability and low cost-of-goods (COGs) required for global therapeutic distribution [13,120,121].

From a strict regulatory perspective, advancing a complex, multi-component cell secretome into human trials necessitates defining clear lot-release criteria [10,11]. Regulatory agencies typically require a validated surrogate potency assay to quantify the biological activity of each commercial batch prior to clinical use [12,122]. Given our extensive in vivo findings demonstrating that FE002-Lu LFCM robustly suppresses TGF-β1 and elevates IL-10, a standardized in vitro assay quantifying these specific cytokine shifts in human macrophage or fibroblast reporter cell lines could serve as a highly reliable additional potency metric [3,122,123]. Of note, recent methodological guidelines emphasize that final lot-release metrics must be translated into primary human immune cells to confirm absolute physiological relevance [122,123]. Establishing this targeted assay early in preclinical development will provide regulatory bodies with the necessary quantitative assurances of batch-to-batch pharmacological consistency.

Finally, future pre-clinical efforts should focus on biodistribution and pharmacokinetic profiling of the nebulized FE002-Lu LFCM formulation to track secretome retention in the human respiratory tree. Given the compelling reduction in mortality-associated fibrotic markers and the suppression of cytokine storms demonstrated herein, lyophilized FE002-Lu LFCM represents a prime candidate for progression into Phase I human clinical trials, offering a highly scalable, potentially disease-modifying intervention for severe pulmonary pathologies [8,55,72].

## 5. Conclusions

Acute LI and its subsequent progression into pulmonary fibrosis represent devastating clinical pathologies characterized by high mortality rates and a current profound lack of curative molecular therapies. The present comprehensive GLP-compliant preclinical investigation demonstrates that FE002-Lu LFCM provides a potent, broad-spectrum therapeutic solution prototype. Across five mechanistically distinct etiologies of pulmonary damage (hyperoxia, LPS, asbestos, silica, and bleomycin), FE002-Lu LFCM consistently worked toward effectively halting disease progression, mitigated structural degradation, and preserved vital alveolar architecture.

The noteworthy efficacy of FE002-Lu LFCM is rooted in its pleiotropic, multi-targeted mechanism of action. During the acute lung injury phase, the secretome effectively suppresses the localized cytokine storm, evidenced by significant reductions in key pro-inflammatory mediators such as IL-1β, IL-6, and TNF-α, and actively stabilizes the delicate alveolar–capillary barrier. Subsequently, it disrupts the chronic fibrotic cascade at its core. Through the robust downregulation of the TGF-β1 signaling axis and pre-translational suppression of α-SMA, FE002-Lu LFCM directly inhibits myofibroblast transdifferentiation and prevents the excessive ECM deposition that drives irreversible scarring. Crucially, the intervention also demonstrates a sophisticated, context-dependent ability to modulate macrophage proteostasis (via MMP-12 and Cathepsin-D); it dampens destructive proteolysis during acute inflammation, while promoting the autophagic turnover and mitigation of newly deposited fibrotic debris during the active remodeling phase.

Ultimately, this study identifies a clear, dose-dependent therapeutic window capable of transitioning the pulmonary microenvironment from a state of advanced, irreversible fibro-proliferation to a regulated, resolving tissue state. Importantly, this study establishes that while mid-tier LFCM dosing provides optimal, balanced protection, secretome saturation at the highest dose in biopersistent particulate models (asbestos) can paradoxically exacerbate fibroproliferation. This finding highlights a critical parameter for future biological dose optimization work. While these 28-day findings in a male rodent cohort establish a robust functional proof-of-concept for early-to-mid-stage intervention, subsequent longitudinal studies incorporating female cohorts will be necessary. These future models must confirm long-term therapeutic durability and evaluate efficacy in late-stage, non-resolving fibrotic environments.

By successfully utilizing a highly stable, lyophilized formulation, this intervention circumvents the stringent cold-chain logistics that currently bottleneck advanced regenerative therapies. This off-the-shelf stability not only presents an ideal candidate for non-invasive, point-of-care aerosolized delivery but also introduces a highly scalable countermeasure suitable for low-resource settings and rapid occupational hazard response. FE002-Lu LFCM’s capacity to successfully target both early inflammatory triggers and chronic remodeling pathways underscores its vast translational potential. Future developmental milestones must now focus on the deep proteomic and transcriptomic deconvolution of this secretome to isolate its active pharmaceutical ingredients, alongside large-animal pharmacokinetic profiling of nebulized formulations. Ultimately, FE002-Lu LFCM stands as a highly promising, disease-modifying prototype poised to address the critical unmet needs in global respiratory medicine.

## Data Availability

The original contributions presented in this study are included in the article/Supplementary Materials. Further inquiries can be directed to the corresponding authors.

## Supplementary Materials

The following supporting information can be downloaded at https://www.mdpi.com/article/10.3390/biomedicines14091888/s1.

## Abbreviations

The following abbreviations are used in this manuscript:

ADAs	Anti-drug antibodies
α-SMA	Alpha-smooth muscle actin
A_260_/A_280_	Absorbance ratio at 260 nm and 280 nm
AECII	Alveolar epithelial type II (cells)
AMMD	Aerodynamic mass median diameter
ANOVA	Analysis of variance
ARDS	Acute Respiratory Distress Syndrome
BALF	Bronchoalveolar lavage fluid
BCA	Bicinchoninic Acid
BLM	Bleomycin sulfate
cDNA	Complementary DNA
CHUV	Centre Hospitalier Universitaire Vaudois
CINC-1	Cytokine-Induced Neutrophil Chemoattractant-1
CO_2_	Carbon dioxide
COGs	Cost-of-goods
C_t_	Cycle threshold
DAB	3,3′-diaminobenzidine
DMEM	Dulbecco’s Modified Eagle Medium
DNA	Deoxyribonucleic acid
DPBS	Dulbecco’s Phosphate-Buffered Saline
DPX	Distyrene, Plasticizer, and Xylene (mounting medium)
ECM	Extracellular matrix
EDTA	Ethylenediaminetetraacetic acid
ELISA	Enzyme-Linked Immunosorbent Assay
EMT	Epithelial-to-mesenchymal transition
EOS	Eosinophils
ET tube	Endotracheal tube
EVs	Extracellular vesicles
FBS	Fetal bovine serum
FiO_2_	Fraction of inspired oxygen
FPCs	Fetal progenitor cells
GAPDH	Glyceraldehyde 3-phosphate dehydrogenase
GLP	Good Laboratory Practice
GMP	Good Manufacturing Practice
GRAS	Generally recognized as safe
H&E	Hematoxylin and Eosin
HCl	Hydrochloric acid
HGF	Hepatocyte Growth Factor
HIER	Heat-induced epitope retrieval
HRP	Horseradish peroxidase
HYP	Hydroxyproline
ICU	Intensive care unit
IHC	Immunohistochemistry
IL-1β	Interleukin-1 beta
IL-6	Interleukin-6
IL-10	Interleukin-10
ILDs	Interstitial Lung Diseases
IND	Investigational New Drug
i.p.	Intraperitoneal
IPF	Idiopathic Pulmonary Fibrosis
i.t.	Intratracheal
LAL	Limulus amebocyte lysate
LC-MS	Liquid chromatography–mass spectrometry
LFCM	Lung fibroblast conditioned medium
LI	Lung injuries
LPS	Lipopolysaccharide
LYM	Lymphocytes
MMP-12	Matrix metalloproteinase-12
MMPs	Matrix metalloproteinases
MON	Monocyte
MSC	Mesenchymal stem cell
NBF	Neutral buffered formalin
NEU	Neutrophils
NF-κB	Nuclear factor-κB
NO	Nitric oxide
OD	Optical density
PBS	Phosphate-buffered saline
PES	Polyethersulfone
PGE_2_	Prostaglandin E_2_
pH	Potential of hydrogen
PLT	Platelets
QoL	Quality of Life
qPCR	Quantitative real-time PCR
qRT-PCR	Quantitative reverse transcription PCR
RIPA	Radioimmunoprecipitation assay (buffer)
RNA	Ribonucleic acid
ROS	Reactive oxygen species
RT	Reverse transcription
SABC	Streptavidin–Biotin Complex
SASP	Senescence-associated secretory phenotype
SD	Standard deviation
SDS-PAGE	Sodium dodecyl sulfate–polyacrylamide gel electrophoresis
SEM	Standard error of the mean
SiO_2_	Silicon dioxide
TBS	Tris-buffered saline
TBST	Tris-buffered saline containing 0.1% Tween-20
TEAC	Trolox Equivalent Antioxidant Capacity
TFF	Tangential flow filtration
TGF-β1	Transforming growth factor-beta 1
TIMP-1	Tissue inhibitor of metalloproteinases-1
TIMPs	Tissue inhibitors of metalloproteinases
TLR4	Toll-like receptor 4
TMB	3,3′,5,5′-Tetramethylbenzidine
TNF-α	Tumor Necrosis Factor-alpha
UDG	Uracil-DNA Glycosylase
*v*/*v*	Volume per volume
WBC	White blood cell
WCB	Working Cell Bank
WISP-1	WNT1-inducible signaling pathway protein-1
