# Supplementary material for: Broad-Spectrum Protective Effects of Lyophilized FE002-Lu Lung Fibroblast Conditioned Medium Against Acute and Chronic Pulmonary Injury in Wistar Rats
Source: Biomedicines. 2026 Aug 24;14(9):1888. doi: 10.3390/biomedicines14091888 (PMC13604320; doi:10.3390/biomedicines14091888)
Supplement: Supplementary file 1 [file biomedicines-14-01888-s001.zip › biomedicines-4479446-supplementary.pdf]

Supplementary Data: Broad-Spectrum Protective Effects of Lyophilized FE002-Lu Lung Fibroblast Conditioned Medium Against Acute and Chronic Pulmonary Injury in Wistar Rats

Lee Ann Applegate *, Alexandre Porcello and Alexis E. Laurent *

Table S1. Significance of color grading for the macroscopic pathological examination of lung tissue.

Color	Primary Driver	Tissue State	Sponginess	Pathology
Reddish	Hemorrhage / Edema	Acute inflammation	Heavy / Wet	Acute alveolitis and congestion
Brownish	Iron (Hemosiderin)	Chronic inflammation	Firm	Hemosiderin accumulation
White/Red	Collagen + Blood	Active scars forming	Irregular / Bumpy	Active fibrogenesis
Whitish	Dense Collagen	Mature fibrosis	Rigid / Rubbery	Mature fibrosis

Table S2. Ashcroft scoring system for the microscopic evaluation of lung fibrosis.

Score	Histological Features
0	Normal lung
1	Minimal fibrosis thickening of alveolar/bronchiolar walls
2	Moderate thickening of alveolar/bronchiolar walls without obvious damage to lung architecture
3	
4	Increased fibrosis with definite damage to lung structure and formation of fibrous bands or small fibrous masses
5	
6	Severe distortion of structures and large fibrous areas
7	
8	Total fibrous obliteration of the field

Table S3. Significance of color grading for the histopathological examination of lung tissue (Picrosirius Red stain).

Parameter	Disease State	Healthy State
Dominant color	Deep red and dark orange	Bright yellow and light orange
Fiber thickness	Thick, dense bundles	Thinner, more delicate network
Alveolar structure	Significant thickening of the septa (walls)	More preserved, thinner alveolar walls
Tissue state	Advanced fibrosis/scarring	Mild injury or normal architecture

Table S4. Details of primer sequences used for qRT-PCR. F, forward primer; R, reverse primer.

Primer No.	Primer Name	Primer Sequence 5' → 3'
1	α-SMA (ACTA2)-F	TTC GCT GTC TAC CTT CCA GC
2	α-SMA (ACTA2)-R	TGG AGG CGC TGA TCC ACA AA
3	TGF-β1-F	TCA GCC AAC AGG AAG GCA TC
4	TGF-β1-R	CAA CCA GCC TCC TAA ACA CCC
5	Collagen-I-F	GAT GCC GGG AGA AAA GGG AA
6	Collagen-I-R	GTC CTG GCG TGC TCA CTA TT
7	Collagen-III-F	GGT CTG AAG GGG TTT CCA GG
8	Collagen-III-R	TCC CAG ACA TTC CCG GTT TG
9	GAPDH-F	CCT GCG ACT TCA ACA GCA AC
10	GAPDH-R	AGG GAG ATG CTC AGT GTT GG

Table S5. Detailed statistics for the results presented in Figure 2. Quantitative modulation of localized and systemic cellular profiles and alveolar inflammatory biomarkers following LFCM treatment. Statistically significant ($p < 0.05$) shifts in leukocyte and platelet populations within both the BALF and peripheral blood, alongside specific pro- and anti-inflammatory mediator concentrations in the BALF. Data for the LFCM-treated cohorts (G3-Low, G4-Mid, and G5-High doses) are compared directly against their respective vehicle-treated disease control groups (G2-Control) across the five study arms. Values are expressed as mean ± SD, with directional arrows (↑/↓) denoting a significant upregulation or downregulation relative to the disease baseline. Concentration units for the inflammatory markers are specified as either pg/mL ([1]) or ng/mL ([2]). BALF, bronchoalveolar lavage fluid; LFCM, lung fibroblast conditioned medium.

Study Arm	Group	Cells in BALF (10^9/L)	Cells in Blood (10^9/L)	Inflammatory Markers in BALF (pg/mL)[1] or (ng/mL)[2]
Hyperoxia	G3-Low dose	/	LYM (↓) [10.71 ± 1.66 vs. 15.44 ± 4.91; $p = 0.046$]	/
	G4-Mid dose	WBC (↑) [0.81 ± 0.66 vs. 0.33 ± 0.16; $p = 0.035$]<br>MON (↑) [0.17 ± 0.16 vs. 0.05 ± 0.06; $p = 0.019$]	/	IL-1β (↓) [75.22 ± 8.07 vs. 120.98 ± 9.72; $p < 0.001$][1]<br>TNF-α (↓) [98.05 ± 11.09 vs. 163.37 ± 24.61; $p < 0.001$][1]<br>NO (↓) [26.35 ± 4.75 vs. 40.63 ± 4.65; $p < 0.001$][2]
	G5-High dose	/	/	IL-1β (↓) [31.57 ± 2.88 vs. 120.98 ± 9.72; $p < 0.001$][1]<br>IL-6 (↓) [58.82 ± 7.07 vs. 165.42 ± 37.90; $p < 0.001$][1]<br>IL-10 (↑) [25.98 ± 6.34 vs. 12.12 ± 2.34; $p < 0.001$][1]<br>TNF-α (↓) [41.23 ± 7.00 vs. 163.37 ± 24.61; $p < 0.001$][1]<br>NO (↓) [21.32 ± 8.41 vs. 40.63 ± 4.65; $p < 0.001$][2]
LPS	G3-Low dose	/	/	/
	G4-Mid dose	/	/	TNF-α (↓) [47.39 ± 20.57 vs. 84.18 ± 16.75; $p < 0.001$][1]
	G5-High dose	/	NEU (↑) [5.24 ± 4.03 vs. 1.91 ± 0.27; $p = 0.006$]<br>MON (↑) [2.00 ± 1.40 vs. 0.97 ± 0.39; $p = 0.034$]	IL-6 (↓) [77.12 ± 18.81 vs. 114.30 ± 10.39; $p = 0.001$][1]<br>IL-10 (↑) [34.90 ± 4.84 vs. 20.79 ± 4.68; $p < 0.001$][1]<br>TNF-α (↓) [29.54 ± 5.78 vs. 84.18 ± 16.75; $p < 0.001$][1]<br>CINC-1 (↓) [78.32 ± 19.00 vs. 158.31 ± 27.50; $p < 0.001$][1]
Asbestos	G3-Low dose	PLT (↓) [63.38 ± 26.45 vs. 124.63 ± 66.10; $p = 0.040$]	WBC (↑) [15.84 ± 3.81 vs. 9.40 ± 3.57; $p = 0.003$]<br>LYM (↑) [12.51 ± 3.20 vs. 7.45 ± 2.77; $p = 0.008$]<br>EOS (↑) [0.21 ± 0.06 vs. 0.11 ± 0.06; $p = 0.023$]	/
	G4-Mid dose	PLT (↓) [64.38 ± 25.99 vs. 124.63 ± 66.10; $p = 0.044$]	WBC (↑) [16.00 ± 4.39 vs. 9.40 ± 3.57; $p = 0.002$]<br>LYM (↑) [12.46 ± 4.30 vs. 7.45 ± 2.77; $p = 0.008$]	IL-6 (↓) [73.62 ± 14.36 vs. 90.10 ± 12.68; $p = 0.048$][1]<br>CINC-1 (↓) [84.41 ± 19.88 vs. 103.62 ± 14.33; $p = 0.030$][1]

			EOS (↑) [0.22 ± 0.05 vs. 0.11 ± 0.06; $p = 0.022$]	
	G5-High dose	/	WBC (↑) [16.37 ± 3.44 vs. 9.40 ± 3.57; $p = 0.002$]<br>NEU (↑) [3.07 ± 1.79 vs. 1.33 ± 0.52; $p = 0.003$]<br>LYM (↑) [12.03 ± 2.68 vs. 7.45 ± 2.77; $p = 0.017$]<br>EOS (↑) [0.24 ± 0.10 vs. 0.11 ± 0.06; $p = 0.018$]	IL-1β (↓) [87.47 ± 12.09 vs. 127.65 ± 27.56; $p = 0.001$][1]<br>IL-6 (↓) [60.21 ± 8.16 vs. 90.10 ± 12.68; $p < 0.001$][1]<br>IL-10 (↑) [18.42 ± 4.94 vs. 13.35 ± 2.71; $p = 0.014$][1]<br>TNF-α (↓) [52.12 ± 17.10 vs. 78.83 ± 8.80; $p = 0.002$][1]<br>CINC-1 (↓) [64.59 ± 14.73 vs. 103.62 ± 14.33; $p < 0.001$][1]
Silica	G3-Low dose	/	/	/
	G4-Mid dose	/	/	IL-6 (↓) [60.41 ± 15.32 vs. 105.77 ± 25.50; $p = 0.002$][1]<br>TNF-α (↓) [54.06 ± 15.37 vs. 83.90 ± 21.54; $p = 0.019$][1]
	G5-High dose	/	/	/
Bleomycin	G3-Low dose	/	/	/
	G4-Mid dose	/	/	/
	G5-High dose	/	NEU (↑) [3.87 ± 2.53 vs. 1.97 ± 1.56; $p = 0.032$]	/

Table S6. Detailed statistics for the results presented in Figure 3. Quantitative modulation of pro-fibrotic mediators, extracellular matrix components, and tissue remodeling effectors in lung tissue homogenates. Statistically significant ($p < 0.05$) alterations in the concentrations of key fibrogenic biomarkers (TGF-β1, TIMP-1, WISP-1) and collagen precursors (hydroxyproline, HYP), alongside the relative protein expression fold-change (FC ratio) of structural and remodeling effectors (α-SMA and MMP-12) assessed via Western blot. Data for the LFCM-treated cohorts are compared directly against the mean baseline values of their respective vehicle-treated disease control groups (G2-Control) across the five study arms. Values are expressed as mean ± SD, with directional arrows (↑/↓) denoting a significant upregulation or downregulation relative to the disease control. Absolute concentrations for the tissue homogenate biomarkers are standardized to total protein and specified as ng/mg ([1]), pg/mg ([2]), or µg/mg ([3]). LFCM, lung fibroblast conditioned medium.

Study Arm	Group	Biomarker Level (ng/mg of protein)[1] or (pg/mg of protein)[2] or (µg/mg of protein)[3]	Biomarker Expression Levels (Protein FC ratio)
Hyperoxia	G3-Low dose	/	/
	G4-Mid dose	TGF-β1 (↓) [10.40 ± 2.01 vs. 18.90 ± 2.58; $p < 0.001$][1]<br>TIMP-1 (↓) [3506.80 ± 263.10 vs. 4139.52 ± 568.00; $p = 0.011$][1]<br>HYP (↓) [2.82 ± 0.15 vs. 4.87 ± 0.71; $p < 0.001$][3]	α-SMA (↓) [0.83 ± 0.96 vs. 4.85 ± 2.28; $p = 0.045$]
	G5-High dose	TGF-β1 (↓) [6.91 ± 2.13 vs. 18.90 ± 2.58; $p < 0.001$][1]<br>TIMP-1 (↓) [2401.58 ± 535.00 vs. 4139.52 ± 568.00; $p < 0.001$][1]<br>WISP-1 (↓) [34.64 ± 4.69 vs. 68.11 ± 5.94; $p < 0.001$][2]<br>HYP (↓) [1.55 ± 0.15 vs. 4.87 ± 0.71; $p < 0.001$][3]	α-SMA (↓) [–0.69 ± 3.56 vs. 4.85 ± 2.28; $p = 0.004$]
LPS	G3-Low dose	/	/
	G4-Mid dose	TGF-β1 (↓) [26.65 ± 8.18 vs. 46.62 ± 19.21; $p = 0.004$][1]<br>HYP (↓) [4.91 ± 1.64 vs. 10.58 ± 3.71; $p < 0.001$][3]	α-SMA (↓) [–1.15 ± 2.63 vs. 1.55 ± 0.70; $p = 0.004$]<br>MMP-12 (↓) [–4.95 ± 7.81 vs. 1.65 ± 0.71; $p = 0.007$]
	G5-High dose	TGF-β1 (↓) [15.16 ± 3.25 vs. 46.62 ± 19.21; $p < 0.001$][1]<br>TIMP-1 (↓) [1942.33 ± 375.00 vs. 2330.10 ± 293.00; $p = 0.026$][1]<br>WISP-1 (↓) [23.64 ± 8.96 vs. 50.12 ± 10.93; $p < 0.001$][2]<br>HYP (↓) [3.25 ± 0.78 vs. 10.58 ± 3.71; $p < 0.001$][3]	/
Asbestos	G3-Low dose	/	/
	G4-Mid dose	TIMP-1 (↓) [2140.82 ± 149.20 vs. 2442.31 ± 242.60; $p = 0.015$][1]<br>HYP (↓) [2.78 ± 0.83 vs. 6.25 ± 1.83; $p < 0.001$][3]	/
	G5-High dose	TIMP-1 (↓) [2049.70 ± 257.80 vs. 2442.31 ± 242.60; $p = 0.001$][1]<br>WISP-1 (↓) [28.39 ± 9.74 vs. 60.15 ± 11.84; $p < 0.001$][2]	/

		HYP (↓) [1.91 ± 0.92 vs. 6.25 ± 1.83; $p < 0.001$][3]	
Silica	G3-Low dose	/	/
	G4-Mid dose	WISP-1 (↓) [27.06 ± 17.32 vs. 52.34 ± 25.46; $p = 0.042$][2]	α-SMA (↓) [–0.21 ± 0.78 vs. 1.90 ± 0.90; $p = 0.003$]<br>MMP-12 (↓) [0.48 ± 1.00 vs. 2.03 ± 1.16; $p = 0.001$]
	G5-High dose	/	α-SMA (↓) [0.06 ± 0.06 vs. 1.90 ± 0.90; $p = 0.009$]<br>MMP-12 (↓) [–0.03 ± 0.18 vs. 2.03 ± 1.16; $p < 0.001$]
Bleomycin	G3-Low dose	/	/
	G4-Mid dose	/	/
	G5-High dose	/	α-SMA (↓) [–0.32 ± 1.37 vs. 1.06 ± 0.42; $p = 0.002$]<br>MMP-12 (↓) [–1.73 ± 4.37 vs. 1.73 ± 0.55; $p = 0.018$]

Table S7. Detailed statistics for the results presented in Figure 4. Quantitative histopathological assessment, structural preservation, and collagen deposition profiles across lung injury models following LFCM treatment. Statistically significant ($p < 0.05$) alterations in pulmonary microarchitecture, structural degradation, and fibrotic tissue distortion. Evaluated parameters include the standardized Ashcroft fibrosis score, morphometric thickness of alveolar walls, alveolar histiocytosis severity, localized collagen deposition scoring, and the total area percentage of Collagen Type I. Data for the therapeutic cohorts (G3-Low, G4-Mid, and G5-High doses) are compared directly against the mean values of their respective vehicle-treated disease control groups (G2-Control) across the five study arms. Values are expressed as mean ± SD, with directional arrows (↑/↓) denoting a significant upregulation or downregulation relative to the disease baseline. Specific parameter measurement types are categorized via superscripts as either area percentages ([1]) or semi-quantitative abnormality/severity scores ([2]).

Study Arm	Group / Dose	Histopathological Parameter (%)[1] or (Abnormality Score)[2]
Hyperoxia	G3-Low dose	Collagen Type-I % (↓) [2.91 ± 4.95 vs. 11.68 ± 7.73; $p = 0.004$][1]
		Thickness of alveolar walls (↓) [1.13 ± 0.35 vs. 1.75 ± 0.46; $p = 0.015$][2]
		Alveolar Histiocytosis (↓) [1.25 ± 0.71 vs. 2.13 ± 0.64; $p = 0.019$][2]
		Collagen Deposition (↓) [1.13 ± 0.35 vs. 1.75 ± 0.46; $p = 0.015$][2]
		Ashcroft scoring (↓) [1.50 ± 1.41 vs. 3.88 ± 1.81; $p = 0.006$][2]
	G4-Mid dose	Collagen Type-I % (↓) [2.61 ± 5.85 vs. 11.68 ± 7.73; $p = 0.003$][1]
		Ashcroft scoring (↓) [1.88 ± 2.10 vs. 3.88 ± 1.81; $p = 0.025$][2]
	G5-High dose	Collagen Type-I % (↓) [2.49 ± 2.08 vs. 11.68 ± 7.73; $p = 0.003$][1]
		Thickness of alveolar walls (↓) [1.00 ± 0.00 vs. 1.75 ± 0.46; $p = 0.003$][2]
		Collagen Deposition (↓) [1.00 ± 0.00 vs. 1.75 ± 0.46; $p = 0.003$][2]
		Ashcroft scoring (↓) [1.13 ± 0.35 vs. 3.88 ± 1.81; $p = 0.001$][2]
LPS	G3-Low dose	/
	G4-Mid dose	Ashcroft scoring (↓) [1.00 ± 0.00 vs. 1.63 ± 0.92; $p = 0.025$][2]
	G5-High dose	Ashcroft scoring (↓) [1.00 ± 0.00 vs. 1.63 ± 0.92; $p = 0.025$][2]
Asbestos	G3-Low dose	/
	G4-Mid dose	/
	G5-High dose	Collagen Deposition (↑) [2.13 ± 0.84 vs. 1.13 ± 0.35; $p = 0.010$][2]
		Ashcroft scoring (↑) [4.50 ± 2.33 vs. 1.38 ± 0.74; $p = 0.007$][2]
Silica	G3-Low dose	Collagen Deposition (↓) [1.50 ± 0.75 vs. 2.38 ± 0.74; $p = 0.029$][2]
	G4-Mid dose	/
	G5-High dose	Collagen Type-I % (↓) [1.37 ± 0.82 vs. 20.10 ± 9.58; $p = 0.004$][1]
		Collagen Deposition (↓) [1.00 ± 0.54 vs. 2.38 ± 0.74; $p < 0.001$][2]
		Ashcroft scoring (↓) [1.88 ± 2.10 vs. 4.50 ± 2.00; $p = 0.035$][2]
Bleomycin	G3-Low dose	Ashcroft scoring (↓) [1.38 ± 0.52 vs. 2.63 ± 1.06; $p = 0.001$][2]
	G4-Mid dose	Ashcroft scoring (↓) [1.13 ± 0.35 vs. 2.63 ± 1.06; $p < 0.001$][2]
	G5-High dose	/

Table S8. Detailed statistics for the results presented in Figure 5. Parallel immunohistochemical and transcriptional profiling of tissue remodeling effectors and fibrillar collagens following LFCM treatment. This table summarizes the statistically significant ($p < 0.05$) alterations in localized protein expression/intensity and pre-translational mRNA expression levels across the five study arms. Spatial protein localization and accumulation are quantified via immunohistochemical (IHC) H-scores. Transcriptional biomarker expression levels are assessed via quantitative reverse transcription PCR (qRT-PCR) and presented as cycle threshold (C_t) values [1]. Data for the LFCM therapeutic cohorts (G3-Low, G4-Mid, and G5-High doses) are compared directly against the mean values of their respective vehicle-treated disease control groups (G2-Control). Values are expressed as mean ± SD, with directional arrows (↑/↓) indicating a statistically significant upregulation or downregulation relative to the disease baseline. LFCM, lung fibroblast conditioned medium.

Study Arm	Group / Dose	Immunohistochemical Parameters (H-Score)	Biomarker Expression Levels (C_t values)
Hyperoxia	G3-Low dose	/	Collagen-I (↓) [22.72 ± 1.46 vs. 25.54 ± 3.10; $p = 0.047$]
	G4-Mid dose	/	Collagen-III (↑) [31.29 ± 1.72 vs. 27.38 ± 1.86; $p = 0.028$]<br>α-SMA (↑) [28.25 ± 2.98 vs. 24.01 ± 2.82; $p = 0.007$]
	G5-High dose	/	Collagen-III (↑) [31.89 ± 4.22 vs. 27.38 ± 1.86; $p = 0.010$]
LPS	G3-Low dose	/	/
	G4-Mid dose	/	α-SMA (↑) [31.46 ± 1.36 vs. 26.40 ± 3.82; $p = 0.017$]<br>TGF-β1 (↑) [26.19 ± 2.14 vs. 22.54 ± 1.64; $p = 0.015$]
	G5-High dose	/	Collagen-III (↑) [28.49 ± 2.77 vs. 23.59 ± 3.93; $p = 0.037$]<br>α-SMA (↑) [32.82 ± 2.31 vs. 26.40 ± 3.82; $p = 0.002$]<br>TGF-β1 (↑) [25.95 ± 3.09 vs. 22.54 ± 1.64; $p = 0.024$]
Asbestos	G3-Low dose	/	Collagen-I (↓) [21.38 ± 1.53 vs. 26.78 ± 4.78; $p = 0.004$]
	G4-Mid dose	/	Collagen-III (↑) [29.60 ± 3.17 vs. 23.47 ± 4.43; $p = 0.016$]
	G5-High dose	α-SMA (↑) [47.25 ± 15.12 vs. 25.50 ± 13.43; $p = 0.013$]<br>Cathepsin-D (↑) [219.17 ± 33.30 vs. 101.00 ± 16.54; $p < 0.001$]	Collagen-III (↑) [31.04 ± 4.15 vs. 23.47 ± 4.43; $p = 0.002$]
Silica	G3-Low dose	/	/
	G4-Mid dose	/	TGF-β1 (↑) [27.96 ± 0.91 vs. 24.52 ± 2.19; $p = 0.018$]
	G5-High dose	/	Collagen-I (↑) [26.10 ± 1.71 vs. 22.30 ± 1.49; $p = 0.001$]<br>TGF-β1 (↑) [29.68 ± 1.76 vs. 24.52 ± 2.19; $p < 0.001$]
Bleomycin	G3-Low dose	/	α-SMA (↑) [31.54 ± 2.47 vs. 24.64 ± 1.02; $p < 0.001$]
	G4-Mid dose	MMP-12 (↑) [83.63 ± 16.31 vs. 50.38 ± 11.72; $p < 0.001$]	Collagen-I (↑) [27.34 ± 1.81 vs. 21.50 ± 1.12; $p < 0.001$]<br>α-SMA (↑) [30.69 ± 3.58 vs. 24.64 ± 1.02; $p < 0.001$]
	G5-High dose	MMP-12 (↑) [84.00 ± 14.02 vs. 50.38 ± 11.72; $p < 0.001$]	Collagen-I (↑) [24.17 ± 1.18 vs. 21.50 ± 1.12; $p = 0.037$]<br>Collagen-III (↑) [30.68 ± 3.09 vs. 22.59 ± 1.04; $p < 0.001$]<br>α-SMA (↑) [31.30 ± 3.13 vs. 24.64 ± 1.02; $p < 0.001$]

[1] An increase in C_t value inversely correlates with target mRNA expression, denoting active pre-translational downregulation of the target gene.

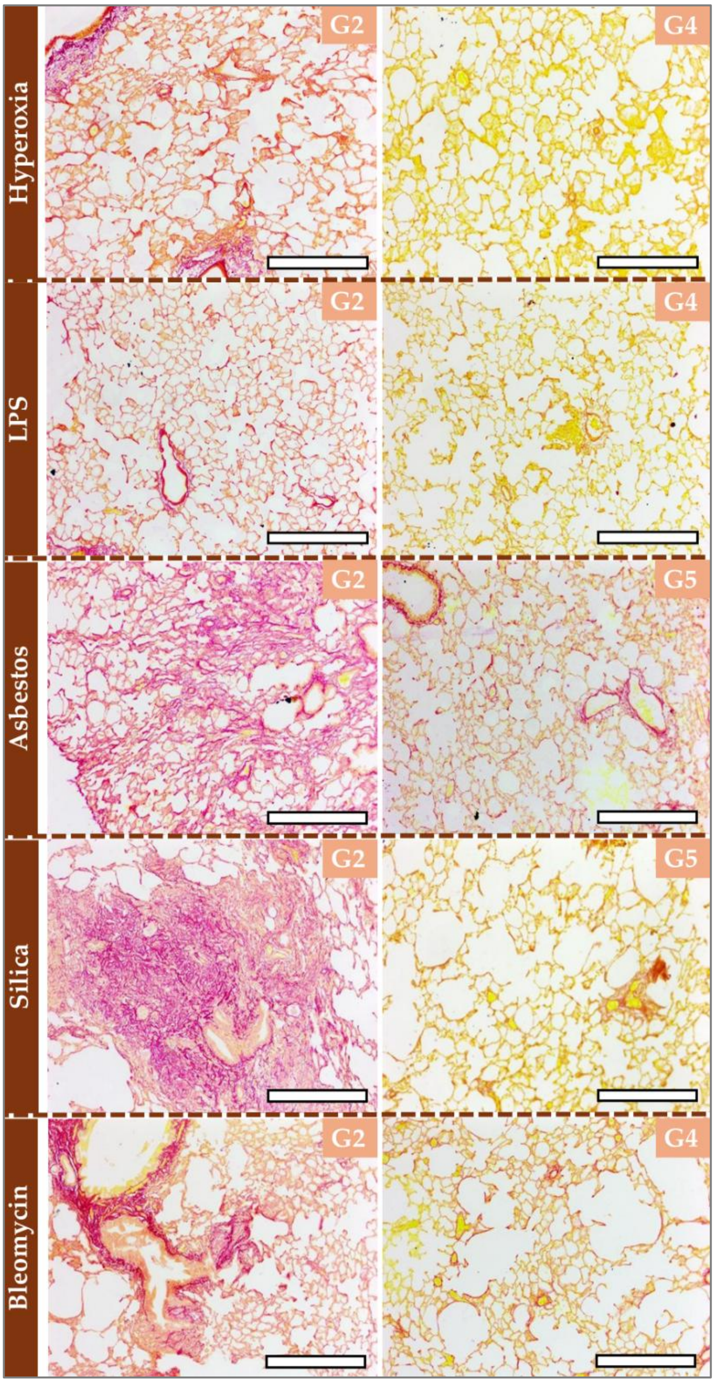

Figure S1. Representative Picrosirius Red stains across study arms and study groups. The G2 positive control groups consistently displayed collagen deposition in alveolar septa (red fibers). Conversely, G4 (LFCM treatment, mid-dose) and G5 (LFCM treatment, high-dose) groups showed limited adverse collagen deposition in alveolar septa. Scale bars = 100 μm. LFCM, lung fibroblast conditioned medium.
